# A medical device-grade T1 and ECV phantom for global T1 mapping quality assurance—the T_1_ Mapping and ECV Standardization in cardiovascular magnetic resonance (T1MES) program

**DOI:** 10.1186/s12968-016-0280-z

**Published:** 2016-09-22

**Authors:** Gabriella Captur, Peter Gatehouse, Kathryn E. Keenan, Friso G. Heslinga, Ruediger Bruehl, Marcel Prothmann, Martin J. Graves, Richard J. Eames, Camilla Torlasco, Giulia Benedetti, Jacqueline Donovan, Bernd Ittermann, Redha Boubertakh, Andrew Bathgate, Celine Royet, Wenjie Pang, Reza Nezafat, Michael Salerno, Peter Kellman, James C. Moon

**Affiliations:** 1 UCL Biological Mass Spectrometry Laboratory, Institute of Child Health and Great Ormond Street Hospital, 30 Guilford Street, London, UK; 2 NIHR University College London Hospitals Biomedical Research Center, Maple House Suite, Tottenham Court Road, London, W1T 7DN UK; 3 Barts Heart Center, St Bartholomew’s Hospital, West Smithfield, London, EC1A 7BE UK; 4 CMR Department, Royal Brompton Hospital, Sydney Street, London, SW3 6NP UK; 5 National Institutes of Standards and Technology (NIST), Boulder, MS 818.03, 325 Broadway, Boulder, CO 80305-3337 USA; 6 Biomagnetics Group, School of Physics, University of Western Australia, 35 Stirling Hwy, Crawley, WA 6009 Australia; 7 NeuroImaging group, MIRA Institute for Biomedical Technology and Technical Medicine, University of Twente, P.O. Box 217, 7500 AE Enschede, Netherlands; 8 Physikalisch-Technische Bundesanstalt (PTB), Abbestr. 2 – 12, D-10587, Berlin, Germany; 9 Cardiology, Charité, Medical Faculty of Humboldt-University Berlin ECRC and HELIOS Clinics, Berlin, Germany; 10 Cambridge University Hospitals NHS Foundation Trust, Cambridge, UK; 11 Department of Physics, Imperial College London, Prince Consort Rd, London, SW7 2BB UK; 12 University of Milan-Bicocca, Piazza dell’Ateneo Nuovo 1, 20100 Milan, Italy; 13 San Raffaele Hospital, Via Olgettina 60, 20132 Milan, Italy; 14 Department of Clinical Biochemistry, Royal Brompton Hospital, Sydney Street, London, SW3 6NP UK; 15 Cardiovascular Biomedical Research Unit, Barts and the London School of Medicine and Dentistry, Queen Mary University of London, London, UK; 16 Resonance Health, 278 Stirling Highway, Claremont, WA 6010 Australia; 17 Department of Medicine (Cardiovascular Division) Beth Israel Deaconess Medical Center, Harvard Medical School, Cardiology East Campus, Room E/SH455, 330 Brookline Ave, Boston, MA 02215 USA; 18 University of Virginia Health System, 1215 Lee St, PO Box 800158, Charlottesville, VA 22908 USA; 19 National Heart, Lung, and Blood Institute, National Institutes of Health, 10 Center Drive, Building 10, Room B1D416, MSC1061, Bethesda, MD 20892-1061 USA; 20 UCL Institute of Cardiovascular Science, University College London, Gower Street, London, WC1E 6BT UK

**Keywords:** T_1_ mapping, Standardization, Phantom

## Abstract

**Background:**

T_1_ mapping and extracellular volume (ECV) have the potential to guide patient care and serve as surrogate end-points in clinical trials, but measurements differ between cardiovascular magnetic resonance (CMR) scanners and pulse sequences. To help deliver T_1_ mapping to global clinical care, we developed a phantom-based quality assurance (QA) system for verification of measurement stability over time at individual sites, with further aims of generalization of results across sites, vendor systems, software versions and imaging sequences. We thus created T1MES: The T1 Mapping and ECV Standardization Program.

**Methods:**

A design collaboration consisting of a specialist MRI small-medium enterprise, clinicians, physicists and national metrology institutes was formed. A phantom was designed covering clinically relevant ranges of T_1_ and T_2_ in blood and myocardium, pre and post-contrast, for 1.5 T and 3 T. Reproducible mass manufacture was established. The device received regulatory clearance by the Food and Drug Administration (FDA) and Conformité Européene (CE) marking.

**Results:**

The T1MES phantom is an agarose gel-based phantom using nickel chloride as the paramagnetic relaxation modifier. It was reproducibly specified and mass-produced with a rigorously repeatable process. Each phantom contains nine differently-doped agarose gel tubes embedded in a gel/beads matrix. Phantoms were free of air bubbles and susceptibility artifacts at both field strengths and T_1_ maps were free from off-resonance artifacts. The incorporation of high-density polyethylene beads in the main gel fill was effective at flattening the *B*_1_ field. T_1_ and T_2_ values measured in T1MES showed coefficients of variation of 1 % or less between repeat scans indicating good short-term reproducibility. Temperature dependency experiments confirmed that over the range 15–30 °C the short-T_1_ tubes were more stable with temperature than the long-T_1_ tubes. A batch of 69 phantoms was mass-produced with random sampling of ten of these showing coefficients of variations for T_1_ of 0.64 ± 0.45 % and 0.49 ± 0.34 % at 1.5 T and 3 T respectively.

**Conclusion:**

The T1MES program has developed a T_1_ mapping phantom to CE/FDA manufacturing standards. An initial 69 phantoms with a multi-vendor user manual are now being scanned fortnightly in centers worldwide. Future results will explore T_1_ mapping sequences, platform performance, stability and the potential for standardization.

**Electronic supplementary material:**

The online version of this article (doi:10.1186/s12968-016-0280-z) contains supplementary material, which is available to authorized users.

## Background

Myocardial tissue characterisation by T_1_ mapping and estimation of extracellular volume (ECV) by cardiovascular magnetic resonance (CMR) is playing an increasingly important role in the diagnosis and management of patients and clinical trials [1]. T_1_ mapping is available as three broad classes of sequences, on multiple platforms, at two field strengths. Factors influencing T_1_ mapping stability and inter-sequence comparisons are well understood [1–4] but little is known about T_1_ mapping delivery at a larger scale over many sites and there is no global quality assurance (QA) system.

The goal of the T1MES program (T1 Mapping and Extracellular volume Standardisation) was to construct an optimised phantom for QA of myocardial T_1_ mapping, covering a relevant range of T_1_ values with suitable T_2_ values for the tissues modelled. The proposed QA consists of regular scans using fixed T1-mapping protocols identical to whatever fixed protocols are used in vivo at each participating site. We therefore aimed for a phantom design that would have stable T1 values for as long as possible. We also aimed for a phantom design avoiding temperature sensitivity of its T1 values as explained later in Methods.

Such a QA system would form part of a system for optimal mapping precision and accuracy [2] within the increasingly known fundamental limitations of the T_1_ mapping methods [5, 6].

The T_1_ Mapping and ECV Standardization (T1MES) program therefore aimed to:

1. Create a partnership of physicists, clinicians and national metrology institutes
2. Design phantom systems for 1.5 T and 3 T for any manufacturer/sequence reflecting T_1_ values in myocardium and blood, pre- and post-Gadolinium-based contrast agents (GBCA)
3. Reproducibly specify and mass produce phantoms with a rigorously repeatable process and to regulatory standards
4. Distribute them to global CMR sites with detailed instructions for fortnightly scanning
5. Publish full details of the formulation to encourage additional applications
6. Measure confounders (e.g. temperature dependency)
7. Analyse scans over 1 year to study the stability of T_1_ measurements over time at each scanner, including a temperature correction model for T_1_
8. Curate phantom data long-term in an open access repository available for reuse/analysis
9. Analyse the inter-site differences in T_1_ values and explore the deliverability of a technique-independent ‘T_1_/ECV Standard’ through local calibration

To date we have achieved steps 1 to 6 of this process, namely the development, testing, certification, QA protocol and preliminary results of T1MES. This paper summarises these first 6 milestones.

## Methods

### Definitions

**Fig. 1:**
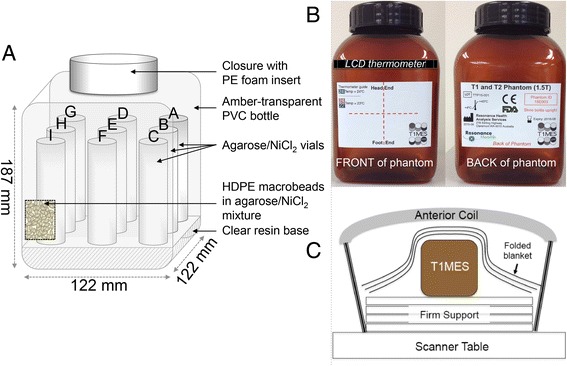
Internal and external phantom structure. Internal (3 T, looking at the front—**a**) and external (1.5 T, front and back—**b**) T1MES phantom structure. The nine tubes are supported on a translucent resin base composed of unsaturated polyester/styrene. A careful hardening and curing process ensured a smooth surface finish for the resin base. The front of the phantom (**b** *left*) contains an isocenter cross label to aid positioning as well as an LCD thermometer. Careful positioning of the bottle on the scanner table (**c**) with the cap towards its head end is needed to ensure it is scanned at isocenter each time. HDPE = high-density polyethylene; LCD = liquid crystal display; NiCl_2_ = Nickel Chloride; PE = polyethylene; PVC = poly vinyl chloride

The term “phantom” refers to the complete test object (Fig. 1).

The term “tube” refers to each of the small bottles embedded within the phantom.

The “gel matrix” is the gel and bead mixture filling the phantom that surrounds all of the tubes.

### Collaboration process

A design collaboration for developing and testing the T1MES phantom and its prototypes was established, consisting of clinicians, physicists, national metrology institutes (the US National Institute of Standards and Technology [NIST] and the German Physikalisch-Technische Bundesanstalt [PTB]) and a small-medium enterprise familiar with phantom production (Resonance Health [RH], Perth, Australia). Funding was secured including a grant from the European Association of Cardiovascular Imaging. Time and expertise was provided for free by the partnership. To engage a global community with constrained funding, the phantoms were gifted (first come, first served) to centers with the proviso that they: a) scan them fortnightly for 1 year and upload the results; b) engage with the partnership to explore any unexpected results; c) do not do anything that could potentially compromise (a) or (b) (e.g. deconstruct the phantom object); and d) give proper reference to the T1MES project if they use the phantoms for other purposes.

### Phantom design

**Fig. 2:**
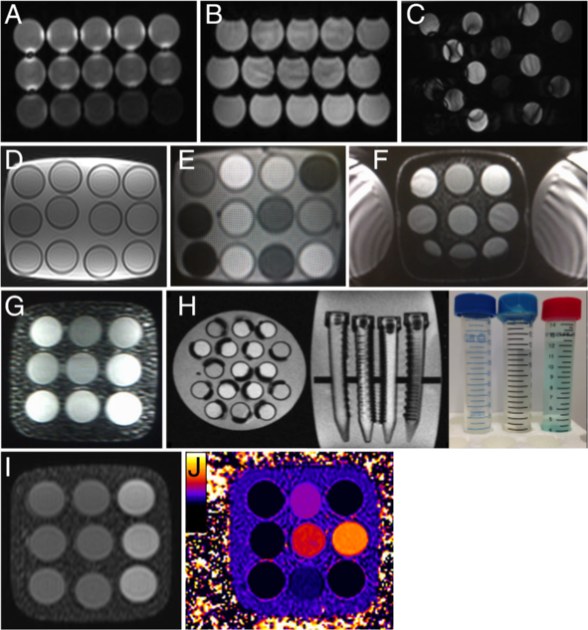
Artifact examples in earlier prototypes (**a**-**g**) and final T1MES phantom (**i**, **j**). Four earlier prototypes (models A—D) were rejected before the final model. **a** Coronal image of the earlier A-model (aqueous fill) showing bright artifacts around the tubes resulting from bSSFP going off-resonance that would have led to variations in T1 values by MOLLI and similar sequences. **b** Transverse image of A-model showing the characteristic ‘cat’s head’ artifact of air-bubbles trapped in the paramagnetically doped aqueous tubes. Significant off-resonance artifact is also noticeable in the central tubes. **c** Another coronal image through A-model but with larger gaps between tubes showing the combined effect of motion artifact (due to the aqueous fill) and *B*_0_ distortion. **d** Transverse image of C-model attempting to use narrower tubes to pack 12 instead of 9, but significant Gibbs artifact can be seen in each tube. **e** Transverse image of C-model showing three small dark circular artifacts (12, 3 and 9 o’clock positions) caused by glue used to stabilize the tube arrangement. We subsequently switched to silicone-based glues that were less likely to trap air bubbles and were artifact-free. **f** Severe stabilisation artifact appearing as a thick dark band around the border of a D-model—here the phantom was scanned immediately after being received from the courier company and the bottle was still very cold from the transportation. Additionally susceptibility artifacts can be seen as thin linear bands spoiling some of the tubes (9 and 3 o’clock). **g** Significant image intensity inhomogeneity during a D-model test session on a GE scanner caused by accidental omission of the folded blanket, intended to separate the phantom bottle from the anterior chest coil. **h** Curved tube artifact and dark rings arising from ink printed onto the sides of digestive tubes (images courtesy of K. E. Keenan and NIST). **i** Coronal bSSFP localiser image and (**j**) typical T_1_ map of a final 3 T T1MES phantom obtained by MOLLI using a bSSFP readout on a Siemens 3 T Skyra scanner. bSSFP = balanced steady-state free precession; MOLLI = modified Look-Locker inversion recovery. Other abbreviation as in Fig. 1

**Fig. 3:**
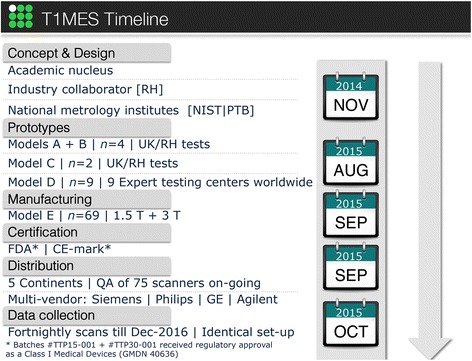
Prototype models and T1MES project timeline. CE = Conformité Européene; FDA = Food and Drug Administration; GE = General Electric; NIST = US National Institute of Standards and Technology; PTB = German Physikalisch-Technische Bundesanstalt; QA = quality assurance; RH = Resonance Health

The design process involved several prototype iterations (known as models A—D before the final mass-production of E-models). Some aspects such as artefacts from the prototype A through D-models that guided the final E-model design are described in Methods and in Fig. 2 with a timeline in Fig. 3. At the very least, the initial A-D models were needed to achieve reasonable T_1_ and T_2_ values without deleterious imaging artefacts, especially as imaging was conducted remotely from the manufacturer.

The range of T_1_ and T_2_ values in the phantom aims to cover typical native and post-GBCA values in both myocardium and blood. The especially wide range of T_1_ post-GBCA (due to variable practice regarding dose, wash-out delays etc. and of course also disease) requires several tubes to cover it. From a review of published values and our own experience, we selected the values listed. Whatever rationale is adopted, with a limited number of tubes there will inevitably be gaps.

T_1_ is generally longer at 3 T compared to 1.5 T. Initially we aimed to design a single phantom for both 1.5 T and 3 T, containing a sufficient number of tubes to cover the needed T_1_ ranges in blood and myocardium, with suitable T_2_ values, pre and post-GBCA at both field strengths. However, the frequency dispersion (i.e. *B*_0_ field dependence) of relaxation times in the phantoms differed strongly from that of myocardium and blood, particularly for the long pre-GBCA tubes, requiring a total of 13 different tubes for 1.5 T and 3 T. Fitting 13 tubes into a single phantom would either have made the object ‘large’ (in relation to the *B*_1_ distortion at 3 T discussed below) or would have required the use of smaller calibre tubes. The following considerations justify our construction therefore of ‘field-specific’ phantoms:

- Tubes had to be a minimum of 20 mm diameter so regions of interest (arbitrarily set to13 mm) would exclude in-plane imaging artifacts at the boundaries between tubes related to the use of clinical T_1_mapping protocols with coarse image resolution, mostly based on single-shot imaging (e.g. Gibbs artifact at the edge of tubes [Fig. 2d] or the potential impact of filtering against it applied differently by various protocol parameters). Altering protocols to optimise phantom scanning would be inconsistent with the aim of the project. The true resolution achieved is further convoluted by the use of asymmetric frequency-encoded readouts for faster repetition time (TR) in balanced steady-state free precession (bSSFP) imaging or partial-phase-encode sampling for shorter total shot duration, and to some extent also by signal variation during the shot.
- Embedding tubes into a gel-filled phantom is important for three reasons: 1) to permit sufficient signal for scanner calibrations; 2) to minimise *B*_0_ and *B*_1_ field distortions local to each tube; and 3) for greater thermal stability. However, embedding all the 13 tubes (to cover 1.5 T and 3 T values) into a single phantom (whether water or water-based gel-filled) will have increased its overall dimensions making it harder to make (our tests and others [7, 8] show that *B*_1_ homogeneity across large ROIs could not be achieved especially at 3 T). Alternative oil-based phantoms have a smaller dielectric permittivity, useful for weaker radiofrequency (RF) displacement current distortion of *B*_1_, but the chemical shift of the matrix fill would require embedded tubes also to use oil-based chemistry (as in diffusion phantoms). Alkanes or similar [9] could not deliver the required range of T_1_ and T_2_ (written as T_1_|T_2_) and a predominately single-peak nuclear magnetic resonance (NMR) spectrum, with the required temperature stability. By using separate water-based gel-filled phantoms for 1.5 T and 3 T with the known high permittivity of water, at a size large enough to fit the needed tubes there was still significant *B*_1_ distortion (range of different flip angles achieved for a prescribed protocol nominal flip-angle) but we were able to counteract it using a method described later.
- This project aims to provide quality assurance for clinically used T_1_ protocols without adapting to the phantom (e.g. no switching to spoiled-gradient echo, or using shorter-TR, no alterations of resolution or field of view etc.; see Additional file 1). Clinical T_1_ mapping protocols are sensitive to off-resonance effects for various well-known reasons. Therefore, *B*_0_distortion near any of the tubes needed to be minimised (tests showed how tube alignment with the *B*_0_ direction was best—this data not shown).

### Phantom materials

All materials proposed for phantoms to date suffer different deficiencies. We adopted the most suitable formulation known, which are paramagnetically doped agarose or carrageenan gels [10, 11]. Some of the main design aspects are listed in Table 1.

**Table 1:** Design factors when developing a T_1_ mapping phantom

Design factor	Explanation	Our proposed solution
Bottle magnetostatics and *B*_*0*_ distortion	The ideal phantom would be uniform and ellipsoidal to avoid susceptibility-induced magnetostatic field perturbation. Such a phantom would permit sphere of Lorentz uniformity but this is not easily mass produced. Many phantoms are cylindrical with the long axis along the static field, *B*_0_ but there is usually off-resonance at the *z*-ends of such objects [7].	An outer phantom body with a smooth surface and soft rounded-edges, placed inside *B*_0_ still distorts some of the imposed magnetic field lines at its *z*-ends so we prescribed scanning halfway along the length of the bottle.
Long term gel stability and risk of moulding	Phantoms with long-term stability could assure the stability of methods applied to patients against scanner alternations and across multiple centers.	Moulding was prevented by aseptic manufacturing, the toxicity of Ni^2+^ ions, and the absence of nutrients in the type of agarose used. Tap water might contain microbial contamination and metal ions so high purity water was used. The main risk is from contraction of gel on loss of water leading to gaps and water condensation but NiCl_2_-doped agarose gel phantoms can be stable over a 1-year period [17].
Seal, leakages, air trapping for aqueous fill	Air pockets in the agarose gel phantom will give rise to susceptibility artifacts on account of the large mismatch in static magnetic susceptibility between air and surrounding gel producing a local distortion in magnetic field strength.	The main phantom was sealed by a black polypropylene screw cap fitted with a polyethylene foam insert. Each internal digestive tube was sealed by a tight screw cap. Gel preparation with warm, degassed water reduced air bubble formation. Note the tube “base-upward” setting procedure and subsequent “top-up” of the contracted gel in each tube after setting, described in the text.
Adjustments of *B*_0_ and reference frequency	Adjustments of *B*_0_ and scanner reference frequency over the phantom have the ability to impact T_1_estimates.	We specified a constant shim volume for each scan. This is manufacturer-dependent—see the T1MES manual [23]. Consistency between repeat scans is the main point.
Gel diamagnetism	In the T1MES model system, because the impact of the paramagnetic ions is so small, we can conceptually treat the main phantom box as if it had no tubes, as if it were just filled with uniform gel throughout	The T1MES system has partly paramagnetic and partly diamagnetic constituents, but the impact of the paramagnetic Ni^2+^ ions is small, around 10 % (because concentrations are small) so the overall interaction is diamagnetic, considering the ~9 parts per million diamagnetism of most tissues relative to air from Lenz electronic diamagnetism.
Gibbs artifact ringing and other inplane effects	Truncating artifacts appear as lines of alternating brightness and darkness in the read-out and phase encode direction. Some effects also from asymmetric readout and *ky* coverage.	Large diameter digestive tubes to house the 9 agarose doped solutions, so that central regions of each tube are sufficiently distant (a number of pixels away) from regions impacted by artifacts from abrupt signal intensity transitions at the tube edges.
1.4 T, 1.5 T, 3 T performance	Many paramagnetic relaxation modifiers, including Mn^2+^ and Cu^2+^, exhibit significant frequency dependence.	We used Ni^2+^[13].
T_1_|T_2_ ranges: blood/myocardium, pre/post-GBCA	The T_1_|T_2_ values were carefully modelled for native and post-gadolinium based contrast agent, blood and myocardium.	5 common tubes, 4 tubes specific to 1.5 T, 4 tubes specific to 3 T. There was no macromolecular addition (no magnetisation transfer modelling) [22].
Tube arrangement	The phantom corners are more prone to inhomogeneities of the *B*_0_ and *B*_1_ magnetic fields.	Longer T_1_ tubes were placed nearer the middle of the phantom layout and avoided the corners.

*Cu* ^*2+*^ copper ions, *Mn* ^*2+*^ manganese ions, *Ni* ^*2+*^ nickel ions, *NiCl*_*2*_ nickel chloride

Agarose or similar gel phantoms are widely used in MR research but less often in commercial phantoms, probably because of long-term stability issues discussed later. Gels permit independent variation of T_1_|T_2_ and they avoid fluid movement within image slice during long inversion recovery (IR) times that could potentially introduce uncertainty in the T_1_* to T_1_ conversion [12]. A more concentrated gelling agent mainly shortens T_2_; a higher paramagnetic ion concentration mainly shortens T_1_ [11, 13]—the two effects are not independent but can be modelled [14] enabling design of mixtures with any required T_1_|T_2_ combination. We did not include sodium chloride (NaCl) (see *B*_1_ uniformity section below). Gel choices include carrageenan, gelatin, agar-agar, polyvinyl alcohol, silicone, polyacrylamide. Some have undesirable NMR spectral properties. The paramagnetic ion choice [15] includes copper, cobalt, iron, manganese (Mn^2+^), gadolinium and nickel (Ni^2+^). Due to the individual T_1_|T_2_ relaxivities of the various ions, no currently known ionic mixture in water can deliver the native myocardial T_1_|T_2_ combination (which requires a relatively high T_1_ with a short T_2_). Ni^2+^ was our first choice as the paramagnetic relaxation modifier at it is less temperature and frequency dependent than other ions [13, 16] and because nickel chloride (NiCl_2_) agarose gel phantoms have been shown to be stable over a 1 year period [17].

### Characterization of T_1_ and T_2_ dependence on agarose and nickel

To achieve the required T_1_|T_2_ tube values we characterised the relation between T_1_|T_2_, agarose and NiCl_2_ concentrations. We made a wide variety of test mixtures as follows: we dissolved at 95 °C for 2.5 h, 135 different concentrations of NiCl_2_, water and agarose, each in a separate 50 ml digestive tube. Using a preheated serological pipette, samples were transferred into preheated NMR tubes (to prevent instant setting of the gel while flowing down the tube), allowed to set and analysed at a measuring temperature of 22 °C with a 1.4 T Bruker Minispec mq60 (60 MHz) relaxometer (Perth, Western Australia). Exponential fitting was done and T_1_ and T_2_ recorded. Based on these results we calibrated the equations [14] modelling the relationship between ingredients and T_1_|T_2_ relaxation times (omitting saline). The model assumes a linear relation between the ingredients and the relaxation rates (R_1_,R_2_) = (1/T_1_,1/T_2_). Using this the ingredients for any required T_1_|T_2_ tube could be calculated. The model was tested for the set of 13 unique T_1_|T_2_ combinations desired for the 1.5 T and 3 T phantoms. Some iterations (models A through D, Fig. 3) were required to derive from the model (based on a non-imaging 60 MHz relaxometer) tube values applicable to clinical 1.5 T and 3 T MR systems described later.

### *B*_0_ uniformity

The approximately cuboid, outer body of the T1MES NiCl_2_-agarose gel phantom (Fig. 1a) consisted of a short, hollow, wide necked and leakproof brown-transparent poly vinyl chloride bottle with a melting temperature of 140 °C (Series #310-73353, Kautex Textron GmbH & Co. KG, Bonn, Germany). The adopted shape is more ellipsoidal than many of the shapes rejected in our tests, consistent with basic magnetostatics (sphere of Lorenz) at 1.5 T and 3 T. The *B*_0_ distortion by the phantom arises from electronic diamagnetism and is not significantly affected by the paramagnetic ion concentrations used. Adding sufficient paramagnetic material to cancel the diamagnetism and flatten *B*_0_ would excessively shorten the relaxation times.

The final body shape gave sufficient *B*_0_ uniformity for T_1_ mapping over only a small region approximately halfway along its length when aligned coaxially with *B*_0_. Regions towards the cap and base of this object were subject to off-resonance errors [18]. The tubes inside the phantom were therefore not fixed directly down to the base of the main bottle. A 20 mm layer of non-coloured (non-saturated) polystyrene resin (Diggers Casting and Embedding Resin 500GM, #FIE00506-9311052000759, Recochem Inc. Perth, Western Australia) was first set hard in the base of the main bottle, and the tubes were adhered to the top of this layer, so that the tubes occupied the middle of the phantom in the cap-to-base direction, where the *B*_0_ field is optimally uniform. *B*_0_ uniformity was mapped to evaluate this cause of distorted T_1_ estimates, using a multi-echo gradient echo sequence based on the phase difference between known echo times [19]. A frequency range of +/−50 Hz across the phantom was regarded as acceptable based on published T_1_-mapping sensitivity to off-resonance [18].

### *B*_1_ uniformity

*B*_1_ uniformity in large water-based phantoms [20, 21] is complex but fundamentally the electric dipole moment of the water molecule rotates in the oscillating electric field associated with the RF *B*_1_field, giving rise to displacement current. Sucrose or other large nonionic molecules can reduce water permittivity, by in effect diluting the problematic water molecules. However, the spectral contribution of such molecules at the high concentrations required is a severe complication. An alternative approach often described in phantom literature is the addition of sodium chloride or similar simple ionic solutes (n.b. not to be confused with high permittivity of powdered titanates, suspended in deuterated water). This tackles the problem from a different direction as it leaves the permittivity unchanged but increases the conductivity (σ) instead, to reduce ωε/σ, i.e. the ratio of displacement current to conduction current. Adding NaCl to the T1MES phantom acted on *B*_1_ distortion at a shallower depth in the T1MES phantom and did not cancel the overall *B*_1_ curvature at any NaCl concentration tested.

In this work, deriving from the sucrose approach, we hypothesised that mixing plastic beads into the matrix gel might also effectively dilute the dielectric permittivity of water and lead to improved *B*_1_ uniformity without directly altering the outer matrix gel T_1_|T_2_ values (see Table 2, 846 ms |141 ms). Our choice of outer matrix gel T_1_|T_2_ values was informed by tests looking at different outer matrix gel T_1_|T_2_ combinations (data not shown) and their impact on bSSFP-stabilisation artifacts at both field strengths. For the beads, two different kinds of plastic bead were evaluated: highly monosized microbeads composed of crosslinked poly methyl-methacrylate (PMMA) polymer (6 μm, Spheromers, Microbeads AS, Norway) and high-density polyethylene (HDPE) beads of oblate spheroidal form (3 mm polar axis by 4.2 mm equatorial diameter) consisting of smooth, semi-translucent, colourless HDPE with a melt index >60 °C (HDPE Marlex HHM 5502 BN, Chevron Phillips Chemical Company LP, Texas, USA). It is important to control the supply of HDPE pellets to ensure that they have not been reground, reblended or otherwise modified. The two different plastic bead versions of T1MES matrix gel were compared to the use of sucrose or sodium chloride (formulations tested: (1) added to 1050 ml of Ni^2+^-doped gelling solution, separately and in combination = 800 g sucrose, 50 g NaCl; (2) added to 1000 ml of distilled water containing NiCl_2_ and MnCl_2_ with T_1_ ~ 600 ms, T_2_ ~ 170 ms: 5 g NaCl; (3) added to 2534 ml of distilled water: 1 g, 4 g, 6.5 g, 11.5 g, 14 g, 19 g, 21.5 g NaCl). *B*_1_ homogeneity was evaluated by flip angle (FA) maps derived by the double angle method using FA 60° and 120° (θ1, 2*θ1) by long TR (8 s) scanning using a 4 ms duration sinc (−3π to +3π) slice excitation width to minimise error due to FA variation through the slice.

**Table 2:** List of T_1_|T_2_ values for the target 13 tubes and outer matrix gel and the required agarose/NiCl_2_ concentrations for the final phantom

Description target (*Tube ID*)	T_1_ (ms at 1.4 T^a^)	T_2_ (ms at 1.4 T^a^)	Agarose (%)	NiCl_2_ (mM)
“Short” post-GBCA blood (*A*)	256	172	0.244	5.547
“Normal” native blood 1.5 T (*B*)	1490	282	0.373	0.362
“Long” post-GBCA blood (*C*)	427	212	0.325	2.860
“Short” native myocardium 1.5 T (*D*)	818	54	2.214	1.231
“Long” native myocardium 1.5 T (*E*)	1384	57	2.279	0.461
“Medium” native myocardium 1.5 T (*F*)	1107	56	2.256	0.725
“Short” post-GBCA myocardium (*G*)	295	50	2.174	4.510
“Long” post-GBCA myocardium (*H*)	557	51	2.377	2.103
“Medium” post-GBCA myocardium (*I*)	429	50	2.306	2.942
“Normal” native blood 3 T (*J*)	1870	288	0.388	0.180
“Short” native myocardium 3 T (*K*)	1043	56	2.245	0.858
“Long” native myocardium 3 T (*L)*	1510	55	2.289	0.342
“Medium” native myocardium 3 T (*M*)	1279	56	2.273	0.531
Outer matrix gel fill	846	141	0.780	1.155

^a^By Bruker minispec mq60 relaxometer 1.4 T (22 °C) at Resonance Health laboratory, Australia

*GBCA* gadolinium-based contrast agents, *ID* identity number

### Temperature dependence of T_1_ and T_2_

Temperature dependency experiments on T_1_|T_2_ values [15] were carried out at various stages:

- **Test 1:** Performed at the PTB laboratory in June 2015 on a 3 T prototype-D (whole phantom with 9 tubes) across 17 temperatures between 14.9 °C and 32.0 °C for T_1_ and across 6 temperatures between 15.6 °C and 31.1 °C for T_2_. Each measurement was repeated twice (with a 2 day gap) and made using a 3 T Siemens Magnetom Verio system (VB17) and a 12-channel head coil.
- **Test 2:** Performed at the NIST laboratory in November 2015 on six loose tubes from the final production run of E-model phantoms. T_1_|T_2_ were measured at 9.9, 17.1, 20.1, 23.1 and 30.1 °C on an Agilent 1.5 T small bore scanner in a temperature-controlled environment. Temperatures were measured using a fiber optic probe. T_1_ was measured by inversion-recovery spin echo (IRSE) (TR [s] = 10, inversion time [TI, ms] = 50, 75, 100, 125, 150, 250, 500, 1000, 1500, 2000, 3000) and T_2_ by SE (TR [s] = 10, TE [echo time, ms] = 15, 30, 60, 120, 240, 480, 960). Note that some of the data acquired under short-term reproducibility was obtained in support of temperature Test 2.

### Short-term reproducibility

Short-term reproducibility (single site, single manufacturer, single sequence) aided temperature sensitivity work and assessed baseline variability between fortnightly scans with all other parameters constant (not least, temperature). For the final T1MES phantom (E-model) two short-term reproducibility experiments were performed:

- **Test 1:** Six loose tubes from the final production run of E-model phantoms were tested for short-term reproducibility of T_1_|T_2_ values at the NIST laboratory in November 2015, at 20.1 °C on an Agilent 1.5 T small bore scanner. T_1_ was measured by IRSE (TR [s] = 10, TE [ms] = 14.75, TI [ms] = 50, 75, 100, 125, 150, 250, 500, 1000, 1500, 2000, 3000) and T_2_ by SE (TR [s] =10, TE [ms] =14.75, 20, 40, 80, 160).
- **Test 2:** One of the final E-model phantoms for 3 T was tested for short-term repeatability of T_1_|T_2_ values using a Siemens 3 T Skyra at Royal Brompton Hospital in November 2015. This test was performed by removing and repositioning the receiver coil, phantom and its supports on each of ten runs, incurring full readjustment of all scanner setup procedures before each run. The acquired data was ten runs, each containing two repeated T_1_ maps, performed at 20.3 ± 0.5 °C. An extension of this work showed that the temperature increase of the T1MES phantom caused by specific absorption rate (SAR) deposition during imaging for repeated T_1_ maps was negligible.

### Detailed construction of phantoms

Some of the detailed construction topics and constraints are listed in Table 1.

Each phantom (1.5 T or 3 T) contains nine tightly capped digestive tubes (#SC475, 50 ml from Environmental Express, South Carolina, USA) embedded in a gel matrix containing Nickel (II) Chloride hexahydrate (99.9999 % purity grade, Acros Organics, New Jersey USA, n.b. highly hygroscopic), high purity deionized water (Ibis Technology) and polysaccharide agarose powder with low endosmotic flow for electrophoresis (molar ratio ≤0.07, Acros Organics).

Mass production was from large batches of 14 solutions (13 tubes + outer matrix gel, Table 2) from which all the tubes and outer containers were filled accordingly. The mass production required some caution against deterioration of the agarose/NiCl_2_ mixtures if kept at high temperatures for periods exceeding around 8 h. The production of all copies of each tube therefore had to be completed within a single working day and as rapidly as possible. Deterioration was noted as a change of agarose gel colour from colourless to faint yellow. Microwave oven heating for initial agarose dissolution was followed by further magnetically-stirred heating and adjustments (based on relaxometry of samples from the mixture). Stirring was essential for uniform gel production into all copies of each tube. Each of the nine tubes is filled with differently doped agarose gels and contains minimal air gaps. Agarose gel contracts as it sets solid, contracting more in stronger agarose mixtures. By “topping up” more gel to the space left by contraction after the initial fill had set in each tube, the air gap can be minimised. Further, by cooling the tubes from the base (by standing them in approximately 2 cm depth of cold water), the gel solidified from the base upward so that contraction left a gap at the top of the tube for adding the “top-up”. This practical step was essential to avoid mid gel contraction gaps forming that is otherwise observed when the gel is allowed to set naturally earlier along the tube sidewalls. Such mid-gel gaps tend to cause a tear down the middle of the gel-filled tube making it unusable for ROI placement in images. The dissolving and solidifying temperatures of agarose gel show hysteresis, dissolving fully only near boiling-point, but requiring cooling to around 45 °C for solidification. The hysteresis assists practically, for example when pouring molten gel around the HDPE beads needed for the main matrix fill.

**Fig. 4:**
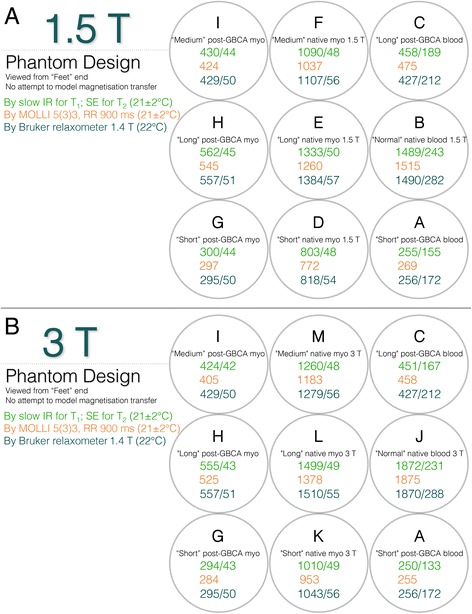
T_1_ and T_2_ values in T1MES. T_1_ and T_2_ values in the phantom mimic those of myocardium and blood pre and post-GBCA at 1.5 T (Panel **a**) and 3 T (Panel **b**). The 13 relaxometry scopes (refer to Table 2) are explained in the figure. Slow scan reference data for T_1_|T_2_ is displayed in green (for T_1_ by slow IRSE and for T_2_ by slow SE, RR interval 900 ms at 21 ± 2 °C), T_1_ values shown in orange represent the mean value per tube derived from tests on five of the E-model phantoms (using a 5(3)3 256-matrix RR = 900 ms at 21 ± 2 °C variant of MOLLI adapted for native T_1_ mapping; Siemens WIP 448B at 1.5 T and WIP 780B at 3 T), and in blue are T_1_|T_2_ values obtained by the manufacturer in Australia using a 1.4 T Bruker minispec relaxometer at 22 °C. Tube arrangement is such that long T_1_ tubes potentially suffering from more artifacts are kept towards the middle of the phantom and away from the corners. GBCA = gadolinium-based contrast agents; IRSE = inversion recovery spin echo; myo = myocardium; RR = inter-beat interval; SE = spin echo. All T_1_|T_2_ values are stated in ms. Other abbreviation as in Fig. 2

**Fig. 5:**
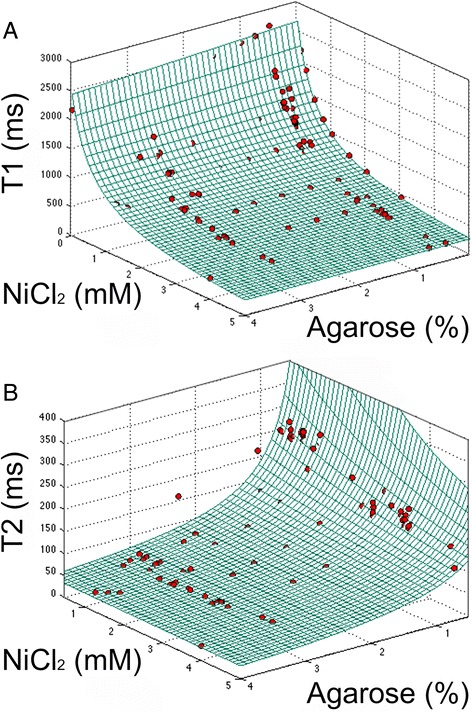
T_1_ and T_2_ relaxation times versus ingredients at 1.4 T: agarose and NiCl_2_. Grid represents results of the model. Red points represent single measurements. **a** Longitudinal relaxation time constant (T_1_), RMSE in R_1_ compared to the linear model was 4.8 × 10^−5^ /ms. **b** Spin–spin relaxation time (T_2_), RMSE in R_2_ compared to the linear model = 5.3 × 10^−4^ /ms. Since the *x* and *y* axes of both fits are comparable, the ingredient that contributes most can be identified. RMSE = root mean square error

Of the 18 tubes used in the 1.5 T and 3 T phantoms, 4 are 1.5 T specific, 4 are 3 T specific (because tissue native T_1_ is longer at 3 T) and five tubes (the post-GBCA tubes) common to both field strengths (Fig. 4). Although some difference in post-GBCA T_1_ values does occur between 3 T vs. 1.5 T, this difference is absorbed within the very wide range of GBCA doses, post-GBCA times, GBCA types etc. in clinical use. Therefore 13 individual recipes were made. The 9 tubes in each field-specific phantom generate 9 different T_1_|T_2_ combinations (Fig. 5) modelled to cover the physiological range of native and post-GBCA, blood and myocardium in health and disease. There was no macromolecular addition with no attempt to model magnetisation transfer [22].

After pouring in the resin base, leaving this to set, and adhering the 9 filled tubes on top of this base using ethylene vinyl acetate and polypropylene uncoloured mixture based hotmelt typically applied from a “hot glue gun”, we packed the compact HDPE pellets into the bottle and then poured in the agarose/NiCl_2_ mixture (typically at a temperature ~ 50–60 °C) taking care to avoid air pockets from forming in the matrix gel fill.

The T1MES phantom has a volume of 2 l, inner length of 187 mm and inner body cross section 122 mm by 122 mm. The labels show an isocenter cross mark, the correct orientation for positioning it under an anterior chest coil, and a serial number and date of manufacture. Also attached to the outside of the phantom is a liquid crystal display (LCD) thermometer of 1 °C resolution. Notably some pigments used on plastic tubes distort the magnetic field [12] (Fig. 2h), so all components were tested carefully, rigorously sourced and documented to avoid unexpected changes which could affect future production batches. Even with the efforts to optimise *B*_0_ and *B*_1_ uniformity, some T_1_|T_2_ combinations are more sensitive to off-resonance errors so these tubes were placed centrally in the phantom avoiding corner locations of greater *B*_0_/*B*_1_ error (explaining the otherwise somewhat counterintuitive ordering of tubes according to their T_1_ values).

Production of one phantom took on average 5 h (distributed over batch production not serial manufacture). As the phantom build was all by manual labour and not automated, it took 3 weeks and four full-time members, 340 h in total to produce the 69 phantoms in this batch.

### Prototype and production batch testing and quality control

Reproducible manufacturing was established for all tubes. Three prototypes (models A to C) had unsatisfactory *B*_0_ and *B*_1_ uniformities before the satisfactory model-D design. Between June and August 2015, 10 D-model phantoms (five for each of 1.5 T and 3 T) were characterized at ten experienced CMR centers for artifacts and for initial verification of the tube T_1_|T_2_ values. In September 2015, the final batch of artifact-free (Fig. 2i, j) T1MES phantoms (E-models) were mass-manufactured and shipped to CMR centers worldwide.

All aspects of phantom production conducted at the RH laboratory were performed in accordance with their certified quality management system including the recruitment and training of staff and the quality control checks of final phantoms. Prior to the mass manufacturing, extensive experiments were done in order to setup the standard operation procedures and working instructions to ensure final phantom integrity. Quality control was ensured at three levels: operator level (e.g. careful choice of materials), engineering level (e.g. the responsible process engineer conducted in-production tests/measurements and inspections, such as checks for bubbles in the tubes and bottle seals, and based on the outcome of this analysis, initiated improvement activities) and management level (e.g. by facilitating training and identifying better measurement or production equipment that could be used for future batches). Operator level quality control evaluated phantoms in real-time during the production process through visual inspection to ensure production ran smoothly, predictably, and to the required standards (e.g. by ensuring a flat resin surface, correctly sealed tubes, tight bead packing of the outer matrix gel, etc.). Overall phantom integrity was also visually checked for any production defects prior to shipment (e.g. precise alignment of isocenter cross label correctly offset from the upper surface of the resin base, no distortion of the outer bottle due to excessively hot gel etc.).

**Fig. 6:**
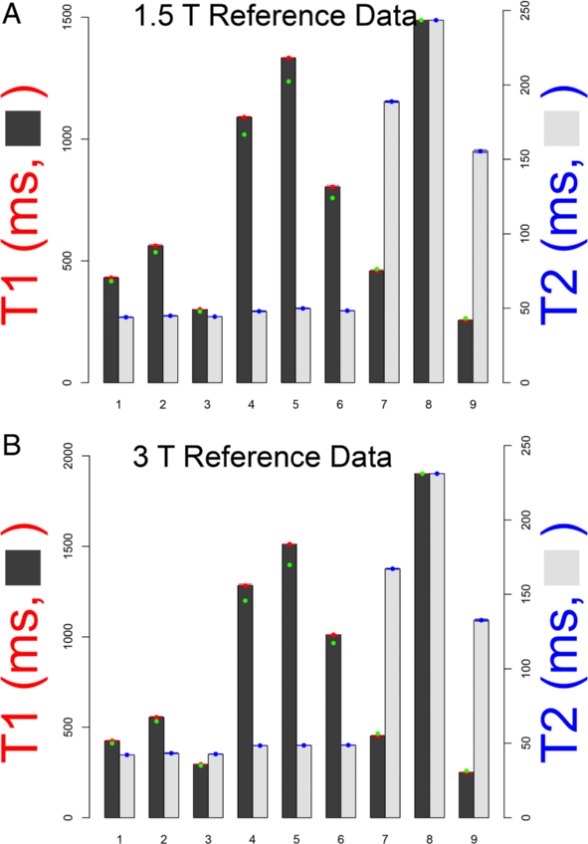
Reference T_1_|T_2_ values. Variation in the mean T_1_ (*red dots*) and T_2_ (*blue dots*) reference values and standard deviation (whiskers) of the nine tubes averaged for the ten final batch T1MES phantoms that underwent ‘gold standard’ slow T_1_ and T_2_ measurements by IRSE and SE respectively at 1.5 T (**a**) and 3 T (**b**). T_1_ values obtained by MOLLI (5(3)3 [256] (WIP# 448B at 1.5 T and WIP# 780B at 3 T) pre-GBCA sequence (*green dots*) are also shown. Abbreviations as in Figs. 2 and 4

Phantom calibration and validation has limitations as phantoms do not fully model tissue (see Discussion). Nonetheless, ‘ground truth’ values in phantoms were measured using slow scanning ‘gold standard’ sequences that have previously demonstrated accuracy in phantom work. Of the 69 final E-model phantoms, 10 (14.5 %; 5 at each of 1.5 T and 3 T) underwent ‘gold standard’ slow T_1_ measurements by IRSE (8 TIs from 25–3200 ms) and T_2_ measurements by slow SE (8 TEs from 10–640 ms) at a single center (Royal Brompton Hospital; Siemens, 1.5 T Aera and 3 T Skyra; Fig. 6). These slow T_1_|T_2_ measurements were only performed once and the results used as ‘ground truth’ for the subsequent measurements. In addition, all tubes were relaxometer-certified pre-assembly.

### Scanning protocol for T1MES

A fundamental aspect of T1MES was to invite each site to submit phantom data with whichever T_1_ mapping sequence they were using clinically. We did not pre-specify any aspect of the T_1_ mapping sequence to use, except careful replication of position and phantom setup without any alteration of the parameters used clinically and not to modify any other parameter of the chosen protocolled T_1_ mapping method during the period of supplying T1MES repeat scans—i.e. to stick to a fixed protocol (as specified in the JCMR Consensus Guidelines for T_1_/ECV). If changes were inevitable, for example due to scanner upgrades, a method of informing T1MES has been implemented and is described in the manual (Additional file 1). Instructions for adjustment and sizing of the shim volume did need to be vendor-specific and these are explained in the appendix section of the T1MES user manual circulated to all participants.

At all participating T1MES sites, the final phantom is currently scheduled for fortnightly scanning for 1 year using a fixed protocol for inter-scan test-retest analysis. Some centers are additionally scanning the phantom using the same sequence at the same position providing data necessary for short-term intra-scan test–retest analysis. Results from this longitudinal data collection are expected to be published in 2017. The T1MES user manual and QA protocol [23] stipulates that the T1MES phantom be kept in the MR magnet room (for stability and also so that its internal temperature will match that displayed by the surface LCD label) and imaged every 2 weeks for 1 year using consistent coil and phantom arrangement. The T1MES user manual emphasises that image parameters be kept unchanged for serial scans except for automatic adjustments of FA and reference frequency. The user manual specifies the range of acceptable positioning of the phantom in the scanner aligned with the main magnetic field. The phantom is scanned axially halfway along the length of the 9 internal tubes corresponding to halfway along the length of the main bottle, imaging only that slice, to avoid *z*-end *B*_0_ distortion. To ensure consistent adjustments of *B*_0_ and scanner reference frequency over the phantom at each repeat scan, the shim volume (also referred to as adjustments volume, adjust region, shim region, shim box) is identically sized and positioned on the phantom bottle for each scan (see Additional file 1). The scan protocol is kept identical for serial scans at each center. Centers were requested to use the same standard anterior chest coil each time.

The minimum fortnightly contribution to T1MES consists of conventional CMR scans: A) the initial localizers; B) at least any one T_1_ mapping sequence with simulated electrocardiogram set at 67 beats per minute (inter-beat [RR] interval 900 ms). The T1MES QA program generates three main types of multicenter data: 1) raw data pertaining to long reference scans for T_1_ (IRSE) and T_2_ (SE) that we reconstruct on receipt: 2) raw T_1_ mapping data from some specific centers without the ability to reconstruct their own maps locally, thus we reconstruct the maps on receipt; 3) reconstructed T_1_|T_2_ maps (majority of sites). T_1_|T_2_ values were taken as mean values from circular ROIs of fixed diameter, in each of the nine tubes in pixel-wise maps.

Within the network are sites using identical magnets, coils and protocols providing an opportunity for a wide range of inter-sequence and inter-site analyses (scheduled for 2017).

### Statistics

Statistical analysis was performed in the R programming language (version 3.0.1, The R Foundation for Statistical Computing). Descriptive data are expressed as mean ± standard deviation except where otherwise stated. Distribution of data was assessed on histograms and using Shapiro-Wilk test. The coefficient of variation (CoV) between repeated scans was calculated as a measure of reproducibility. For defining the model that describes the relation between ingredients and relaxation rates (R_1_|R_2_), the fitted parameters were found by fitting a surface for both T_1_ and T_2_ using the MATLAB (The MathWorks Inc., Natick, MA, USA, R2012b) curvefitting tool and the linear least-squares approach. The analysis of incoming T1MES datasets is carried out using a MATLAB graphical user interface. From the data, mean T_1_ and T_2_ values were measured from each of the nine contrast tubes. Using the ROI measurement tool in MATLAB, mean signal intensity of the central 50 % area of each of the nine tubes was calculated.

## Results

### Model predictions of T_1_ and T_2_

Linear models for longitudinal and transverse relaxation rates R_1_|R_2_ in terms of the ingredients agarose and NiCl_2_ can be written following similar work previously published [14]:

$$ {R}_x/{\mathrm{ms}}^{-1} = {a}_x+{b}_x\ {C}_{w, agarose}/\%+{c}_x{C}_{{\mathrm{Ni}}^{2+}}/\ \mathrm{m}\mathrm{M} $$

where *x* = 1, 2, *C*_*w,agarose*_ and ${C}_{{\mathrm{Ni}}^{2+}}$ are the weight and molar concentration of agarose and Ni^2+^, respectively, and *a*_*x*_, *b*_*x*_ and *c*_*x*_ are found by surface fitting (Fig. 5):

$$ {a}_1 = 3.750\times {10}^{-4},\kern0.5em {b}_1 = 8.790\times {10}^{-6},\kern0.5em {c}_1 = 6.683\times {10}^{-4} $$

$$ {a}_2 = 1.645\times {10}^{-4},\kern0.5em {b}_2 = 7.622\times {10}^{-3},\kern0.5em {c}_2 = 7.201\times {10}^{-4} $$

From these relationships and replacing relaxation rate *R*_*x*_ by relaxation time *T*_*x*_ we calculated the required agarose % (by weight) and Ni^2+^ concentrations (equal to added molar concentration of NiCl_2_.6H_2_O as it is highly dissociated) for each of the 13 tube stock solutions as shown in Table 2.

The presented model was accurate within the root-mean-square errors (RMSE) in Fig. 5 caption over the range T_1_ = 300–1900 ms and T_2_ = 40–300 ms that cover the range of relaxation times expected in healthy and diseased myocardium pre- and post-GBCA.

### Reference T_1_ and T_2_ values

Comparison of ‘gold standard’ T_1_ and T_2_ values (Fig. 6) between the ten E-model phantoms tested, confirmed reproducibility of manufacturing. Across the 9 tubes, CoV for T_1_ ranged from 0.17 to 1.25 % at 1.5 T and 0.08 to 1.0 % at 3 T, while T_2_ ranged from 0.74 to 2.12 % at 1.5 T and 0.40 to 1.72 % at 3 T.

### *B*_0_ uniformity

**Fig. 7:**
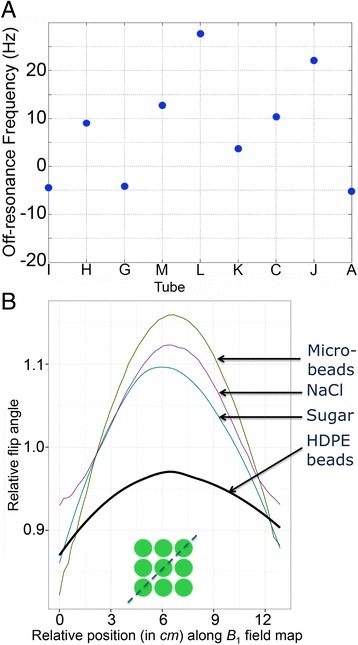
*B*_0_ and *B*_1_ field homogeneity. **a** *B*_*0*_ field homogeneity across the nine phantom compartments as a measure of off-resonance in Hz at 3 T (single E-model phantom results). These are extremely small shifts in frequency (30 Hz = 0.25 ppm) at 3 T and should not be regarded as significantly different between the tubes. **b** Diagonal profile of the *B*_1_ field (as per *green* discontinuous line in the inset) comparing relative flip angles on a Siemens 3 T system. Variance of *B*_1_ was smallest across the 9 compartments with CoV 1.54 % for HDPE beads consisting of smooth, semi-translucent, colourless compact discs (as colouring in plastics has the potential to distort the *B*_0_ magnetic field [12], see Fig. 2h) with a melt index >60 °C. We choose pellets that had not been regrinded, reblended or composite for this purpose. Highly monosized microbeads measured 6 μm and were composed of crosslinked PMMA polymer. Neither microbeads, sucrose nor NaCl were comparably effective in flattening the *B*_1_ field. PMMA = poly methyl methacrylate. Other abbreviation as in Fig. 4

Final phantoms were free of air bubbles and susceptibility artifacts at both field strengths. T_1_ maps were obtained in the specified mid-phantom slice at the specified scan setup, and were free from off-resonance artifacts (Fig. 2i, j). Provided the bottle was placed coaxial with *z*-axis, imaged as a transverse slice halfway along, and with the use of shimming as specified in the T1MES manual, *B*_*0*_ uniformity was delivered (Fig. 7a) to within ±30 Hz at 3 T.

### *B*_1_ uniformity

The compact HDPE beads (~1 kg of compact pellets per phantom bottle) adequately flattened the *B*_1_ field at 3 T (Fig. 7b), compared to the PMMA microbeads, sucrose and sodium chloride. The HDPE beads cause a speckle of dark regions in the gel matrix as they generate no MR signal that is normally detectable. The beads are expected to have similar diamagnetism to the gel so they have no impact on the *B*_0_ field.

### Temperature dependency experiments

**Fig. 8:**
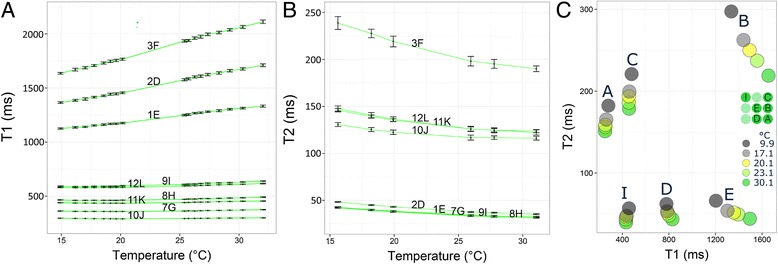
Temperature experiments in T1MES. Temperature dependency experiments (Test 1 in methods) performed on a D-model whole phantom (tube nomenclature differed from that used in E-models) comparing the stability of T_1_ (**a**) and T_2_ (**b**) values between two repeat experiments (2 days apart) at various temperatures between 15 °C and 32 °C on a 3 T Siemens Verio system. Whiskers represent mean ± standard error. (**c**) Temperature dependency experiment (Test 2 in methods) comparing T_1_|T_2_ values in tubes A, B, C, D, E and I (*middle right insert*) from a final E-model phantom across five temperatures

Collectively the results (Fig. 8) by slow SE scanning methods show that over the range 15–30 °C the short-T_1_ tubes are more stable with temperature than the long-T_1_ tubes where T_1_ increased more strongly with temperature. T_2_ values also change significantly with temperature (Fig. 8b), decreasing as temperature increases.

### Short-term reproducibility

**Fig. 9:**
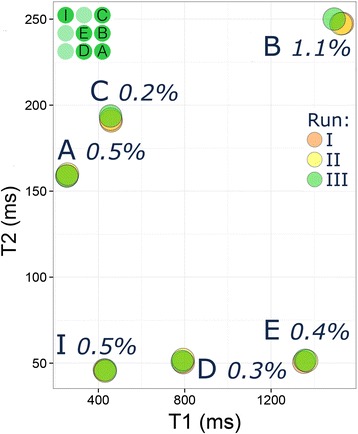
Short-term reproducibility. Short-term reproducibility (three runs) at the NIST laboratory (Test 1 in methods) for phantom T_1_values in six loose tubes (*top left insert*) from a final E-model phantom showing CoV of 1 % or less. Tube B with the longest T_1_|T_2_ showed the greatest variability between reads. CoV = coefficient of variation
- **Test 2:** Test-retest evaluation of one of the final phantoms for 3 T by cardiac T_1_ mapping, including complete repositioning and readjustments, also gave a short-term repeatability CoV for T_1_ ≤1 % (Table 3 detailing results for 3 T). For T_2_ measured by fast T_2_-prepared single-shot methods, the CoV was usually below 1 % with an exceptionally large 4.1 % in the tube B with longest T_1_.

- **Test 1:** Six loose tubes as used in the 1.5 T E-model (Fig. 9) showed a CoV of ≤1 % for both T_1_ and T_2_reproducibility. Tube B with the longest T_1_ and T_2_ showed the greatest variability between repeated scans.

**Table 3:** Short-term reproducibility experiments in a 3 T final phantom (E-model)*

Tube	Parameter	Sequence	CoV (%)	Mean diff. ± s.d.
A	T_1_	pre_MOLLI_5(3)3_256_T1	0.16	255 ± 0.4
		post_MOLLI_4(1)3(1)2_256_MOCO_T1	0.18	255 ± 0.5
	T_2_	T2_4pt_TRUFI_192i_T2	0.66	194 ± 1.3
		T2_4pt_GRE_192i_T2	0.61	134 ± 0.8
J	T_1_	pre_MOLLI_5(3)3_256_T1	0.14	1860 ± 2.6
		post_MOLLI_4(1)3(1)2_256_MOCO_T1	0.17	1672 ± 2.8
	T_2_	T2_4pt_TRUFI_192i_T2	4.06	227 ± 9.2
		T2_4pt_GRE_192i_T2	1.37	203 ± 2.8
C	T_1_	pre_MOLLI_5(3)3_256_T1	0.08	460 ± 0.4
		post_MOLLI_4(1)3(1)2_256_MOCO_T1	0.08	461 ± 0.4
	T_2_	T2_4pt_TRUFI_192i_T2	0.52	195 ± 1.0
		T2_4pt_GRE_192i_T2	0.76	160 ± 1.2
K	T_1_	pre_MOLLI_5(3)3_256_T1	0.13	953 ± 1.2
		post_MOLLI_4(1)3(1)2_256_MOCO_T1	0.10	917 ± 0.9
	T_2_	T2_4pt_TRUFI_192i_T2	0.98	60 ± 0.6
		T2_4pt_GRE_192i_T2	0.67	49 ± 0.3
L	T_1_	pre_MOLLI_5(3)3_256_T1	0.08	1372 ± 1.1
		post_MOLLI_4(1)3(1)2_256_MOCO_T1	0.16	1252 ± 2.0
	T_2_	T2_4pt_TRUFI_192i_T2	0.91	56 ± 0.5
		T2_4pt_GRE_192i_T2	0.89	49 ± 0.4
M	T_1_	pre_MOLLI_5(3)3_256_T1	0.15	1178 ± 1.8
		post_MOLLI_4(1)3(1)2_256_MOCO_T1	0.12	1104 ± 1.3
	T_2_	T2_4pt_TRUFI_192i_T2	0.91	58 ± 0.5
		T2_4pt_GRE_192i_T2	0.66	49 ± 0.3
G	T_1_	pre_MOLLI_5(3)3_256_T1	0.19	285 ± 0.6
		post_MOLLI_4(1)3(1)2_256_MOCO_T1	0.20	285 ± 0.6
	T_2_	T2_4pt_TRUFI_192i_T2	0.29	86 ± 0.2
		T2_4pt_GRE_192i_T2	1.02	49 ± 0.5
H	T_1_	pre_MOLLI_5(3)3_256_T1	0.11	527 ± 0.6
		post_MOLLI_4(1)3(1)2_256_MOCO_T1	0.09	527 ± 0.5
	T_2_	T2_4pt_TRUFI_192i_T2	0.35	66 ± 0.2
		T2_4pt_GRE_192i_T2	0.72	46 ± 0.3
I	T_1_	pre_MOLLI_5(3)3_256_T1	0.06	406 ± 0.3
		post_MOLLI_4(1)3(1)2_256_MOCO_T1	0.05	409 ± 0.2
	T_2_	T2_4pt_TRUFI_192i_T2	0.21	72 ± 0.2
		T2_4pt_GRE_192i_T2	0.19	47 ± 0.1

*All tests performed at 20.3 ± 0.48 °C on Siemens, Skyra 3 T at RBHT, November 2015 with RR interval 900 ms and using two T_1_ mapping sequences (pre-MOLLI 5(3)3 [256] and post-MOLLI 4(1)3(1)2 [256] with MOCO, WIPs# 780B) and two T_2_ mapping sequences (TRUFI T2 map and GRE T2 map)

*CoV* coefficients of variation, *diff.* difference, *GRE* gradient echo, *MOCO* motion correction, *MOLLI* modified Look-Locker inversion recovery, *RR* inter-beat interval, *s.d.* standard deviation, *TRUFI* true fast imaging with steady-state free precession

### Production, distribution and initiation of trial

On 1st September 2015 the E-model T1MES phantoms (batch numbers TTP15-001 and TTP30-001 for 1.5 T and 3 T respectively) received regulatory clearance by the Food and Drug Administration (FDA) and Conformité Européene (CE) marking as a Class I Medical Device (GMDN 40636). This initial mass manufacturing phantom experience was not always 100 % successful and important quality control lessons have been learnt: for example two different fill solutions for tubes were accidentally mislabelled initially and had to be discarded and remade; individual tubes with visible bubbles on inspection had to be corrected with appropriate procedures; any solution stock with T_1_ or/and T_2_ not falling within +/− 3 % of our pre-specified targeted range had to be adjusted.

A total of 75 multi-vendor CMR scanners (four systems: Siemens, Philips, General Electric [GE] and Agilent) across five continents (Table 4), are currently using T1MES phantoms for their local T_1_ mapping QA as part of the international T1MES program. This amounts to an initial 53 individual CMR centers and 69 devices, with six centers using the same field-specific phantom for QA scans on more than one local machine.

**Table 4:** Quality assurance of T_1_ mapping: the initial T1MES CMR centers

Center	Magnet characteristics						
	Vendor	Tesla	Name	YOM	Software	Bore^b^ (cm)	Gradient performance^c^
St Thomas’ Hospital UK	Siemens	1.5	Aera	2015	VE11	70	45/200
St Thomas’ Hospital UK	Philips	1.5	Ingenia	2013	R4.1.3SP2	70	33/200
Oslo University Hospital Norway	Siemens	1.5	Aera	2014	VE11	70	40/200
Bristol Heart Institute UK	Siemens	1.5	Avanto	2009	VB17A	60	44/180
Diagnostikum Berlin Germany	Siemens	1.5	Aera	2015	VE11	70	45/200
GOSH UK	Siemens	1.5	Avanto	2007	VB17	60	40/180
NIH Bethesda US	Siemens	1.5	Aera	2014	VE11	70	45/200
Pittsburgh Pennsylvania US	Siemens	1.5	Espree	2009	VB17A	70	40/200
Leiden UMC The Netherlands	Philips	1.5	Ingenia	2014	R5.1.7SP2	70	45/200
Leeds General Infirmary UK	Philips	1.5	Ingenia	2014	R5.1.7SP2	70	45/200
MUMC The Netherlands	Philips	1.5	Ingenia	2012	R 5.1.7SP2	70	45/200
Policlinico San Donato Italy	Siemens	1.5	Aera	2012	VD13A	70	45/200
Papworth UK	Siemens	1.5	Avanto	2008	VB17A	60	50/200
Wythenshawe Manchester UK	Siemens	1.5	Avanto	2008	VB17A	60	45/200
Copenhagen University Hospital Denmark	Siemens	1.5	Avanto	2008	VD13A	60	45/200
Queen Elizabeth Hospital Birmingham UK	Siemens	1.5	Avanto	2008	VB17A	60	33/125
Birmingham Children’s Hospital UK	Siemens	1.5	Avanto	2010	VB17A	60	33/125
University of Kentucky USA	Siemens	1.5	Aera	2012	VD13A	70	45/200
Charles Perkins Sydney Australia	Siemens	1.5	Avanto	2013	VE17A	70	45/200
Taichung Veterans Hospital Taiwan	Siemens	1.5	Aera	2005	VE11	60	45/200
Monash Heart Australia	Siemens	1.5	Avanto	2010	VB17	55	40/200
Niguarda Hospital Milan Italy	Siemens	1.5	Avanto	2005	VB17A	60	40/200
Golden Jubilee Glasgow UK	Siemens	1.5	Avanto	2008	VB17A	60	45/200
T-T!ME Multi-center phantom^a^							
INSERM U1044 France	Siemens		Aera	2012	VD13A	70	40/200
King Abdul-Aziz Saudi Arabia	GE	1.5	Discovery MR450	2012	DV24	60	50/200
Prince Charles Hospital Queensland	Siemens	1.5	Aera	2011	VD13A	70	45/200
Federal Medical Center Moscow	GE	1.5	Optima MR450w	2014	DV25	70	44/200
Medical University of Vienna Austria	Siemens	1.5	Avanto	2006	VD13B	60	40/200
DHZ Berlin Germany	Philips	1.5	Achieva	2008	R5.1.8	60	33/180
St George’s University London UK	Siemens	1.5	Aera	2014	E11	70	45/200
RBHT London UK	Siemens	1.5	Avanto	2005	VB17A	60	40/170
University Hospital Southampton UK	Siemens	1.5	Avanto	2006	VB17A	60	40/200
Barts Heart Center London UK	Siemens	1.5	Aera	2014	VD13A	70	45/200
Barts Heart Center London UK	Siemens	1.5	Aera	2015	VE11A	70	45/200
The Heart Hospital London UK	Siemens	1.5	Avanto	2009	VD13A	70	40/200
Charité Campus Buch Germany	Siemens	1.5	Avanto	2007	VB13B	60	40/200
University of Virginia US	Siemens	1.5	Avanto	2005	VB17A	60	45/200
University of Virginia US	Siemens	1.5	Avanto	2015	VD13A	60	45/200
SIEMENS EU	Siemens	1.5	Aera	2009	VE11	70	45/200
UZ Leuven Belgium	Philips	1.5	Ingenia	2007	R5.1.7	60	45/ 200
UZ Leuven Belgium	Philips	1.5	Achieva XR	2014	R5.1.7	70	33/122
Beth Israel Deaconess Medical Center, US	Philips	1.5	Achieva	2005	R3.2	60	33/180
NIH Bethesda US	Siemens	1.5	Aera	2012	VD13A	70	45/200
St Thomas’ Hospital UK	Philips	3	Achieva TX	2007	R3.2.3	60	40/200
St Thomas’ Hospital UK	Siemens	3	Biograph mMR	2013	VB20P	60	45/200
Fondazione Toscana Monasterio Pisa Italy	Philips	3	Ingenia	2012	R5.1.8	70	45/200
Oslo University Hospital Norway	Philips	3	Ingenia	2011	5.1.7	70	45/200
Oslo University Hospital Norway	Siemens	3	Skyra	2014	VE11	70	45/120
CRIC Bristol UK	Siemens	3	Skyra	2009	VD13C	60	44/180
Diagnostikum Berlin Germany	Siemens	3	Skyra	2012	VE11	70	45/200
University of Aberdeen Scotland UK	Philips	3	Achieva TX	2015	R5.1.7	60	80/100
NIH Bethesda US	Siemens	3	Verio	2009	VB17	70	33/125
Leiden UMC The Netherlands	Philips	3	Achieva TX	2012	R5.1.8.2	70	45/200
MUMC The Netherlands	Philips	3	Achieva TX	2011	R 3.2	60	40/200
Wythenshawe Manchester UK	Siemens	3	Skyra	2014	VE11	70	45/200
Copenhagen University Hospital Denmark	Siemens	3	Verio	2010	VB17	70	45/200
Charles Perkins Sydney Australia	GE	3	Discovery MR750w	2014	DV25	70	44/200
BHF Glasgow Center UK	Siemens	3	Prisma	2015	VE11	60	80/200
INSERM U1044 France	Siemens	3	Prisma	2015	VE11	60	80/200
DHZ Berlin Germany	Philips	3	Ingenia	2011	R5.1.8	70	45/200
St George’s University London UK	Philips	3	Achieva TX	2012	R5.1	60	40/150
RBHT London UK	Siemens	3	Skyra PTX	2011	VD13C	70	43/180
Barts Heart Center London UK	Siemens	3	Prisma	2015	VE11	60	80/200
Leeds General Infirmary UK	Philips	3	Achieva TX	2010	R5.2	60	40/120
Montreal Heart Institute Canada	Siemens	3	Skyra	2012	VD13A	70	45/200
PTB Germany	Siemens	3	Verio	2010	VB17A	70	45/200
University of Virginia US	Siemens	3	Skyra	2011	VE11A	70	45/200
UZ Leuven Belgium	Philips	3	Ingenia	2010	R5.1.7	70	45/200
NIH Bethesda US	Siemens	3	Skyra	2012	VD13A	70	45/200
University of Queensland Australia	Siemens	7	Magnetom 7	2013	VB17B	60	72/200
University of Queensland Australia	Siemens	3	Trio TIM	2008	VB17A	60	45/200
Glenfield Hospital Leicester UK	Siemens	3	Skyra	2010	VD13A	70	45/200
Baker IDI Australia	Siemens	3	Prisma	2014	VD13D	60	80/200
NIST US^d^	Agilent	1.5	Varian	2013	VnmrJ 4	14	300/475
NIST US^d^	Agilent	1.5	Varian	2013	VnmrJ 4	14	300/475

^a^This phantom is a gift to support the ongoing ’T-T!ME’ study. It will be scanned across multiple UK centers

^b^Inner diameter i.e. around patient

^c^Maximum gradient performances as returned on the T1MES registration forms by each site. These values are subject to many modifying conditions. More relevant parameters such as TR and TE will be extracted from uploaded Digital Imaging and Communications in Medicine (DICOM) images where this is possible from DICOM

^d^Loose tubes only for 1.5 T and 3 T

*YOM* year of manufacture

## Discussion

Results obtained thus far demonstrate that: 1) mass production of phantoms to regulatory standards and in accordance with a rigorously repeatable process is feasible, 2) based on the sequences used, T_1_|T_2_ times in gels are highly reproducible in the short-term, 3) a significant temperature dependency of measured T_1_|T_2_ values exists in tubes with longer T_1_ values that will require the use of a correction model.

The T1MES program seeks to advance the field of quantitative CMR relaxometry and the use of imaging biomarkers like T_1_ mapping and ECV in clinical trials and clinical practice. Our aim was to collaborate with industry, with leading CMR academics and clinical centers with an interest in T_1_ mapping, so as to develop and test a multicenter QA infrastructure, to protect normal reference data at centers and also potentially to improve consistency of T_1_ mapping and ECV results across imaging platforms, clinical sites, and over time. Key to the achievement of accurate and reproducible T_1_mapping/ECV results in CMR is the accelerated development and adoption of rigorous hardware and software standards.

However, this proposal is subject to a further limitation that the phantoms do not model other aspects of tissues, particularly for myocardium—the magnetisation transfer [22] neither does it address the mapping techniques’ ability to discriminate T_1_ values between adjacent regions of interest (the clinical challenge of discriminating tissue T_1_ values in adjacent myocardial segments). For example, the signal-to-noise ratio in the phantoms is unrealistically high as the surface coils are typically nearer; evaluating such an ability is beyond the scope of T1MES. The only realistic aim may prove to be that of providing individual (or genuinely identical) centers with a QA phantom that could protect normal reference data and assure (or even permit correction of changes in) stability of protocols during a long study.

The 1-year study, now running, is expected also to give information about gel stability. It seems reasonable to expect sudden steps in T_1_ values from genuine changes in the acquisition, or scatter from any remaining uncontrolled parameters or imperfect temperature correction, but there would be a gradual monotonic drift as the gel water content changes. Agarose gel is inherently unstable even within a sealed tube, because the gel contracts as water leaves it, appearing as excess water (as droplets) in the gap left by the contraction, often visible on the inner wall of the tube. Note that this effect can occur within well-sealed tubes. It is unrelated to contamination because agarose without added nutrients does not support mould growth. Over time, this shrinkage may also occur in the matrix fill leading to air-gaps and *B*_0_ distortion, potentially occurring near the tubes making a possible contribution to an apparent drift in T_1_ values over time. For the first time, the 1-year study will give large-scale initial data on the durability of this type of phantom. At study end, we aim to recall approximately 10 % of the phantoms which will be inspected for flaws in the gel using high-resolution 3D imaging, with collection also of long reference T_1_|T_2_data as gel drying with shrinkage and condensation into the gap is known to occur even within a sealed tube. Centers are free to keep and use the T1MES phantoms after the 1-year study ends. There is no provision for return shipment to the coordinating site, nor any knowledge of how long the gels will remain usable.

The field and temperature dependence of T_1_ for phantoms containing Ni^2+^ is much smaller than those containing other paramagnetic ions like Cu^2+^. As T_1_ increases above 500 ms (in tubes with a low concentration of Ni^2+^), the tube’s T_1_ becomes more temperature-sensitive as it is increasingly dominated by the temperature sensitive T_1_ of water in the gel [24, 25]. Therefore temperature monitoring of each fortnightly session is essential. Our results enable us to integrate a temperature-correction model into our multicenter T1MES analysis, that will be published at the end of the project. The temperature sensitivity of T_1_ revealed in the present work may not be a concern for clinical T_1_ mapping in healthy volunteers (as the human body is homeothermic—temperature of 37 °C) but it may be a concern for hypothermic or febrile patients. Furthermore T_2_ temperature dependence could also impact measured T_1_ as some fast-T_1_ methods have considerable T_2_ sensitivity.

## Conclusion

We report on the establishment of a collaboration to develop CMR phantoms to CE/FDA standards and an initial multicenter repeat scanning program aiming for global QA of T_1_ and ECV protocols. A rigorous and reproducible manufacturing process for the phantoms has been established. The temperature sensitivity, short-term stability and inter-phantom consistency have all been assessed in support of the main project. An initial 69 phantoms with a multi-vendor user manual are now being scanned fortnightly in centers worldwide, permitting the academic exploration of T_1_ mapping sequences, platform performance and stability over a year.

## Additional file

Additional file 1: The T1MES User Manual. (PDF 13350 kb)

## Abbreviations

CE: Conformité Européene
CoV: Coefficients of variation
Cu^2+^: Copper ions
DICOM: Digital imaging and communications in medicine
ECV: Extracellular volume
FA: Flip angle
FDA: Food and Drug Administration
GBCA: Gadolinium-based contrast agents
GE: General electric
HDPE: High-density polyethylene
Hz: Hertz
IRSE: Inversion recovery spin echo
LCD: Liquid crystal display
Mn^2+^: Manganese ions
MOLLI: Modified look-locker inversion recovery
MR: Magnetic resonance
NaCl: Sodium chloride
Ni^2+^: Nickel ions
NiCl_2_: Nickel chloride
NMR: Nuclear magnetic resonance
PMMA: Poly methyl-methacrylate
QA: Quality assurance
R_1_|R_2_: Relaxivity of T_1_ and T_2_
RF: Radiofrequency
RMSE: Root-mean-square error
ROI: Region of interest
RR: Inter-beat interval
SAR: Specific absorption rate
SASHA: Saturation recovery single-shot acquisition
SE: Spin echo
ShMOLLI: Shortened modified Look-Locker inversion recovery sequence
T_1_|T_2_: T_1_ and T_2_
TE: Echo time
TI: Inversion time
TR: Repetition time
