# Supplementary material for: A medical device-grade T1 and ECV phantom for global T1 mapping quality assurance—the T1 Mapping and ECV Standardization in cardiovascular magnetic resonance (T1MES) program
Source: J Cardiovasc Magn Reson. 2016 Sep 22;18:58. doi: 10.1186/s12968-016-0280-z (PMC5034411; doi:10.1186/s12968-016-0280-z)
Supplement: Additional file 1: — The T1MES User Manual. (PDF 13350 kb) [file 12968_2016_280_MOESM1_ESM.pdf]

THE T1MES PHANTOM USER MANUAL

CONTACTS FOR ADVICE:

Dr Peter Gatehouse
p.gatehouse@rbht.nhs.uk
Cardiac MRI Department,
Royal Brompton Hospital,
Sydney Street,
London, UK SW3 6NP

Prof James C Moon
j.moon@ucl.ac.uk
Barts Heart Center,
West Smithfield,
London, UK EC1A 7BE

Dr Gaby Captur
capturgaby@hotmail.co.uk
Barts Heart Center,
West Smithfield,
London, UK EC1A 7BE

T1MES cmr
T1 Mapping and ECV Standardisation
Improving patient care through CMR phantoms

Manufacturer:
Resonance Health,
278 Stirling Highway,
Claremont, Australia WA 6010
support@resonancehealth.com

European Authorised Representative:
Medical Device & QA Services Ltd.
Spring Court, Spring Road,
Hale, Cheshire, UK WA14 2UQ

ADDITIONAL MATERIALS NEEDED TO SCAN THE PHANTOM

1. A **firm support** to elevate the T1MES bottle to isocenter (for approximate heights please see the specific **Siemens/Philips/General Electric (GE) Appendix** scanner instructions but these may vary depending on couch options).
2. A **double folded blanket** to separate the phantom from the anterior coil.
3. **For GE centers only** – a 'Chicken Heart' ECG Simulator.

Written and produced by Gatehouse P, Captur G, Moon JC, Pang W, Royet C.
Every effort shall be made to keep this manual as relevant and up-to-date as possible – so it is subject to change without notification. Version 27 October 2015.

Regulatory information – Medical Device classification:

**Europe**	**USA**
Class I non-sterile and non-measuring<br>CE	Class I<br>FDA Establishment Registration #3005450718

TABLE OF CONTENTS

ABBREVIATIONS LIST **2**

INTRODUCTION TO THE T1MES USER MANUAL **3**

1 | PHANTOM OVERVIEW **4**

2 | PHANTOM CARE **5**

3 | INSTRUCTIONS FOR PHANTOM SETUP **5**

3.1 | TEMPERATURE *5*

3.2 | ORIENTATION *6*

4 | PHANTOM SCANNING **7**

4.1 | SCAN SETUP *7*

5 | SHIM SETTINGS AND SHIM VOLUME **9**

6 | ONLINE DATA TRANSFER **10**

6.1 | REDCap Module *10*

6.2 | DICOM PORTAL *11*

7 | RESULTS **11**

8 | APPENDIX **12**

*A8.1 | **SIEMENS**-specific instructions* *12*

*A8.2 | **PHILIPS**-specific instructions* *14*

*A8.3 | **GE**-specific instructions* *16*

ABBREVIATIONS LIST

AP = antero-posterior
ECG = electrocardiogram
FOV = field of view
GE = General Electric
HF = head-foot
ID = phantom identity number
LCD = liquid crystal display
$NiCl_2$ = nickel chloride
PMU = physiologic monitoring unit
P(P)NS = predicted peripheral nerve stimulation
RL = right-left
SAR = specific absorption rate
T = tesla
T1MES = T1 Mapping and ECV Standardisation

INTRODUCTION TO THE T1MES USER MANUAL

Above all, the aim is consistency each time the phantom is setup and measured. These instructions mainly attempt to keep everything the same every time: phantom orientation and position, coil setup, heart rate, factors affecting scanner calibrations such as the “shim” volume, shim methods, etc. Please try to keep this basic aim in mind. Although running the B_0 “shim” is arguably undesirable please note this is specified and should be operated in the same mode with the same receiver coils for it, and the same calculation or fitting volume used each time.

The arrangement of tubes in the phantom is not random: it avoids placing long-T1 tubes in the corner positions where B_0 and B_1 distortions associated with the phantom are greatest. Alignment of tubes with the B_0 direction and scanning halfway along them, not towards their ends, are also important factors in avoiding measurements in regions using distorted fields.

Please do not add “loading” phantoms – the phantom has been tested without these on all systems.

The suggested R-R interval 900ms (heart-rate 67bpm) requires explanation. For good reasons known in the literature, some T1-mapping methods on some scanners run for a fixed period of seconds, fitting in however many images they can, at one per cardiac-cycle in the prescribed time. However, a phantom scan using a simulator resulting in an exact "fit" of cycles into the prescribed delay, could result in a variable number of images caused by small simulator timing differences on repeated scans. The R-R at 900ms is an attempt to avoid this problem in the most common case (Siemens 5s[3s]3s and 4s[1s]3s[1s]2s). Depending on your T1-mapping sequence, an R-R of 800ms or 1000ms may still run repeatably reliably. Provided that the SAME R-R value is used for each session this would be acceptable if 900ms cannot easily be obtained (but if possible please use 900ms).

1 | PHANTOM OVERVIEW

1. The phantom consists of an amber plastic bottle sealed by a tight black cap.

   △ **NEVER OPEN THE CAP**

2. Inside the bottle is an agar/nickel chloride ($NiCl_2$)/high-density plastic bead fill containing a 3 X 3 array of plastic tubes filled with the T1/T2 mixtures. The tubes rest on a resin layer at the base (**Figure 1**).
3. There is a label with a red isocenter cross on the front surface of the bottle to aid positioning in the scanner.
4. There is another label on the back of the bottle that contains the unique 6-digit phantom identity number (ID) - e.g. 15E001 or 30E008. The first 2 numbers (‘15’ | ‘30’) indicate whether this is a 1.5T or 3T phantom. ‘E’ indicates the model, and the last three digits are the unique serial number.
5. Please use the Phantom ID for all data entry.
6. Along the top of the bottle’s front surface, is a liquid crystal display (LCD) thermometer strip.

Figure 1. External features of the phantom.

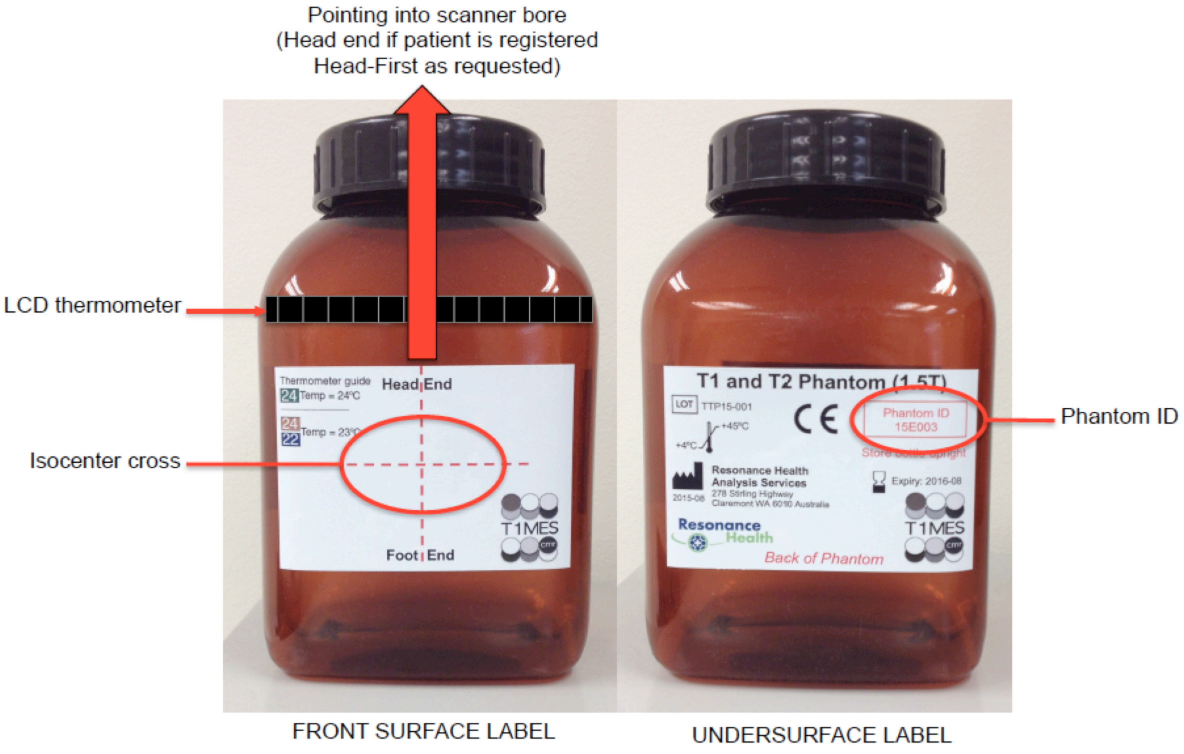

2 | PHANTOM CARE

1. On receiving the phantom, unpack and inspect it. Take photos if it appears damaged and upload these onto REDCap (see 6.1). Use REDCap to log a description of the fault.
2. Please be gentle when moving the phantom around - do not drop or shake it.
3. Do not place heavy objects on top of or strongly against the phantom at any time (the phantom outer bottle is quite flexible).
4. In between research scans, store the phantom upright in your scanner room for temperature consistency with scanning, and in the same protected area each time.

△ **STORE THE PHANTOM VERTICALLY UPRIGHT**

3 | INSTRUCTIONS FOR PHANTOM SETUP

3.1 | TEMPERATURE

1. There is a self-adhesive LCD temperature strip with temperature range 10°C to 40°C along the upper surface of the phantom (**Figure 2**). Temperature markers appear at an interval of 2°C, but the **resolution of the strip is actually 1°C**.
2. Take note of phantom temperature **before** starting each new scan.
3. If there is a bright GREEN cell, this marks the exact phantom temperature (it will be an even number).
4. If there is no green cell but adjacent BLUE and TAN cells, then exact phantom temperature is the odd number **in between** (**Figure 3**).
5. Phantom temperature must be added to the <Patient Name> when **registering the new scan** on your MRI scanner (see 4.1).

Figure 2. Temperature strip

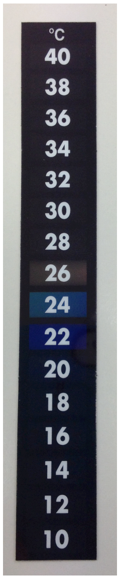

Figure 3. If **GREEN** cell = take this value;
If adjacent **TAN/BLUE** cells = take middle value.

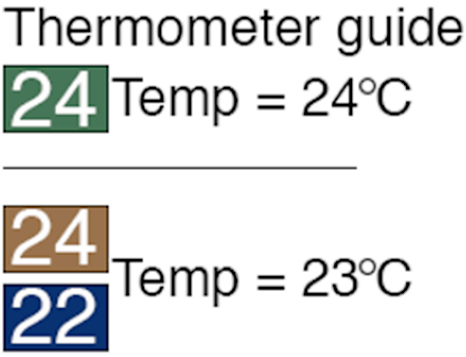

3.2 | ORIENTATION

△ AIM TO POSITION THE PHANTOM AT ISOCENTER USING THE SAME SUPPORTING MATERIALS IN EACH SCANNING SESSION
(+/-1cm across [*x*] and along [*z*] the bed; +/-3cm for height [*y*])

1. Set up and scan the phantom at the isocenter position and in exactly the same way each time for repeat scans.
2. If you notice something unspecified while handling/scanning the phantom, please feedback on what it was.
3. Read your manufacturer-specific instruction in the **Appendix** for where along the table to place the phantom, i.e. in the *z* direction (for Siemens see A8.1.1; for Philips see A8.2.1; for GE see A8.3.1).
4. Do not place the phantom directly onto the spine or bed coil. To bring it up to isocenter you must elevate the phantom bottle above the spine or bed coil by using a firm support.
5. This height depends on your scanner model and MRI table options. (For Siemens see A8.1.1; for Philips see A8.2.1; for GE see A8.3.1). Test the height of the firm support to find one that will lift your phantom to isocenter (see **Figure 4**).

△ USE THIS SAME FIRM SUPPORT FOR ALL SUBSEQUENT SCANS.

6. The firm support must be **FIRM**. It cannot be so soft that it will deform erratically and prevent consistent phantom positioning (i.e. avoid ordinary pillows and blankets for this application).
7. Suggested materials to use as firm support include:
   a. A stack of glued (not stapled) spine journals.
   b. A firm supporting (foam plastic) slab that may come with your scanner equipment.
8. Once you configure the correct firm support STORE it in the scanner room with the phantom.
9. Lay the T1MES phantom onto the firm support as in **Figure 4**.

△ STORE AND ALWAYS USE THE SAME FIRM SUPPORT FOR EACH SCAN

Figure 4. Phantom setup on the table (the 'M' of T1MES in the figure below is at isocenter).

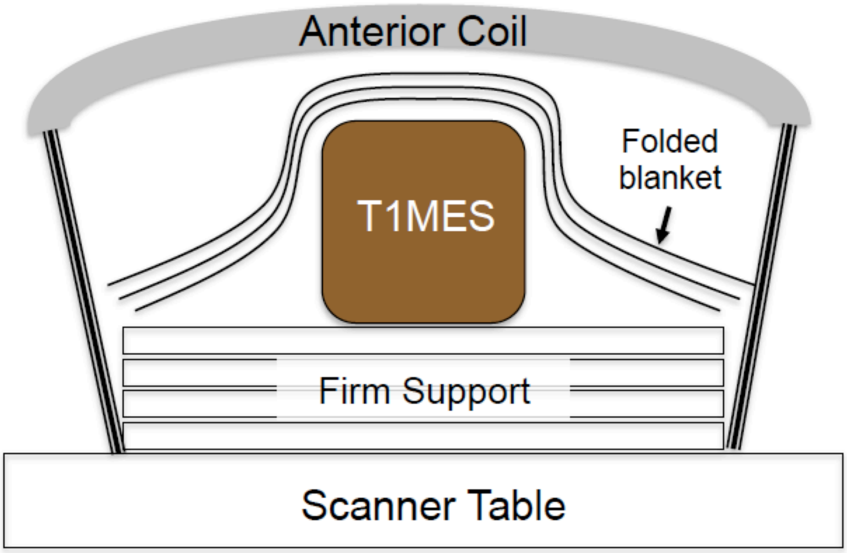

10. Consistent elevation will optimise the height of T1MES isocenter so that you obtain repeated T1MES data with CONSISTENT tube position and orientation over time.

11. Place the firm support in the middle of the table's receiver coil relative to *x*-axis and between the correct table markers for where you would usually position the patient's heart.
12. The **black bottle cap** (equivalent to the '**Head**') for the specified head-first registration of patient orientation must point into the bore. The isocenter cross sticker must be on the upper surface of the bottle.
13. ***Before doing anything else***, move the table and shine the laser target markers onto the phantom arrangement till the laser markers are aligned with the red isocenter cross. The pair of *x*-lines and the pair of *z*-lines should overlap.
14. **Memorize phantom temperature now and before introducing bottle into the bore**.
15. Obtain a thick blanket, folded over into a generous rectangular shape and drape this over the top of the phantom. Do this ***after*** having isocentered with lights, ***but before*** applying the surface coil.
16. If possible, use the same anterior cardiac coil for all subsequent phantom experiments.
17. During the subsequent steps, note that the phantom may accidentally be shifted or twisted, and this **should be avoided**.
18. Place the anterior coil onto the blanket that is overlying the phantom with laser target marker still turned on. Adjust the position of the anterior coil manually (do not move the table nor inadvertently slide the phantom) till the ***isocenter cross of the coil*** is aligned with the laser target markers.
19. Strap the anterior coil into place to prevent it slipping off the phantom arrangement. Tighten the straps to secure the blanket around the T1MES bottle.
20. While unlikely to matter, please use consistent air-flow settings for repeated scans (e.g. **set the patient fan in the scanner to medium** each session).
21. Once you configure the correct firm support, STORE it in the scanner room ready for repeated use with the phantom.

4 | PHANTOM SCANNING

4.1 | SCAN SETUP

1. Register the phantom on your MRI scanner using the Phantom ID. For Siemens see A8.1.2; for Philips see A8.2.2; for GE see A8.3.2.
2. **FOR THE MAJORITY OF CENTERS** that are scanning **one phantom on just one magnet** serially for one year please register the phantom as follows:

   Enter **Temperature after** the <Phantom ID> and **before** the <Date> (DDMMYYYY) separated by underscores.

   E.g. if the bottle is 15E001 and its temperature is 22°C at the start of the scan conducted on 31st January 2015, then log scan as:

   15D001_22_31012015

3. **FOR THE MINORITY OF CENTERS** that are scanning **one phantom on more than one magnet** serially for 1 year please register the phantom as follows:

   After the <Phantom ID> enter the '3-letter' <**Magnet Denomination**> that we will provide for each of the magnets you intend to use. Enter **temperature after** the <Magnet Denomination> and **before** the <Date> (DDMMYYYY) separated by underscores.

E.g. if the bottle is 15E001 and its temperature is 22°C at the start of the scan conducted on 31st January 2015 on the 1.5T Philips Achieva XR, then log scan as:
15D001axr_22_31012015

AND

E.g. if the same bottle 15E001 is of temperature 23°C at the start of the scan conducted on 3rd February 2015 on the 1.5T GE Signa HDx, then log scan as:
15D001sdx_23_03022015

4. Set up the simulated electrocardiographic (ECG) signal. Please see the note in the <Introduction> explaining the 900ms advised and when, if necessary, other R-R intervals could be used. For Siemens see A8.1.3; for Philips see A8.2.3; for GE see A8.3.3.
5. Before starting the scans make sure **all relevant coils are switched on.**
6. Start with scout or localizer imaging. For simplicity it is best if these localisers are all **acquired at isocenter with no image-plane or field of view (FOV) offsets away from isocenter**. Note that: **preset localiser protocols often contain such FOV-shifts or offsets** as typically suiting average patient morphology in the registered patient orientation. It may therefore be easier if you **TURN OFF/UNCHECK** any such FOV-shifts/offsets before running the scout.
7. Check that the physical positioning of the T1MES phantom is correct (along all three directions) as follows and that the phantom is not tilted or twisted (for system specific instructions on this see Appendix).
8. Isocentering the T1MES phantom along the head-foot (HF) direction (red lines in **Panels A** and **B** in **Figure 5**) should simply be governed by the laser marker. Similarly, isocentering the bottle along the supine patient right-left (RL) direction (yellow lines in **Panels B** and **C** in **Figure 5**) should again simply be governed by the laser marker.
9. Using the firm support, the T1MES phantom should be at the correct **height** such that the isocenter is halfway up the central tube, and at the level of the blue line in **Panels A** and **C**, **Figure 5**.
10. In **Panel C** (**Figure 5**) along the yellow line (i.e. height [*y*]), if the isocenter is further than **3cm** from the center of the middle tube, then **positioning is not acceptable**. Please pull out the table, ***change the height of the firm support*** and try again. Once you obtain the correct support for your scanner/phantom arrangement remember this and if possible **store the materials used with the phantom**, so that repeated scanning is less painful!
11. In the other two directions (along the patient bed [*z*] and across the patient bed [*x*]) the center of the middle tube should be **≤1cm** from isocenter. This should be easy to achieve using the laser guidelines and isocenter cross.
12. Please check that the phantom is not tilted or rotated in any axis beyond 10 degrees.

Figure 5. Achieving perfect isocenter position of T1MES phantom.
Note that this does not depict any particular scanning software and is for guidance in interpreting the isocenter graphics in use on your particular machine.

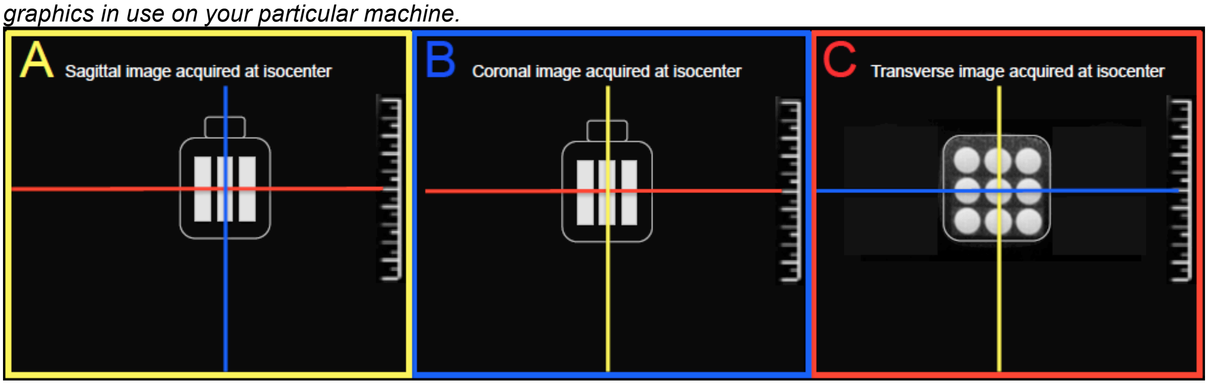

5 | SHIM SETTINGS AND SHIM VOLUME[1]

△ **BE CAREFUL TO SET THE SHIM VOLUME AS SPECIFIED IN THIS MANUAL**

1. Before running any T1 mapping sequence you must select the correct shimming method and specify a particular shim volume over the T1MES phantom. Please follow the manufacturer-specific instructions for these steps (see the Appendix).
2. Apply the same shim volume before starting each set of experiments in a reproducible manner.
3. The shim volume should be positioned at isocenter.
4. **Remember to wait at least 10 seconds with no scanner activity whatsoever before running the first T1 map on your phantom bottle**.
5. Now run your T1 mapping sequence positioned transversely and positioned with FOV centered exactly at isocenter in all three directions.
6. **Remember to wait at least 10 seconds with no scanner activity whatsoever before running any further T1 map on your phantom bottle**.
7. If multiple T1 sequences are run, please try to apply them in the same order each time. This is simply to help us with data processing not for physics reasons. A typical site would run just 1 or 2 versions each time the bottle is scanned (e.g. a MOLLI and a ShMOLLI). Running several repetitions of a T1 sequence each time would also be welcome for some indication of any short-term scatter (or serious drift issues!). Please tell us if you change these sequences during the repeated supply of T1MES data by completing a REDCap module.
8. If you have access to both the <pre-GAD> and <post-GAD> versions of a particular T1 mapping sequence, we would be grateful if you could run both on the phantom each time, with suitable definite scanner inactivity before each (10 seconds if in doubt!). Running both <pre> and <post-GAD> T1 mapping sequences is **not** obligatory - we leave it up to each center to provide what they can repeatedly over the 1-year period.
9. Do not modify the FOV or any other parameters of your chosen protocolled T1 mapping during the period of supplying T1MES repeat scans – stick to a fixed protocol (as specified in the JCMR Guidelines for T1/ECV). If you deviate from a protocol for any given reason or are forced to change sequence please you will need to complete a REDCap module for that scan date.

[1] *N.B. 'Shim Volume' = shorthand for adjustments volume, adjust region, shim region, shim box. See manufacturer-specific Appendix for more details.*

6 | ONLINE DATA TRANSFER

6.1 | REDCap Module

1. Near shipping time you will have received a further REDCap survey from us, collecting more detailed site-specific information.
2. You will then receive an email with account details to be able to access the REDCap online **T1MES_Project** database. You may wish to write down login details here:

   USERNAME ________________________________

   PASSWORD________________________________

3. To access REDCap enter this URL in your browser:

   https://redcaphh.c-cloudservices.net/

4. Click on <My Projects> to find the T1MES_Project.
5. Having opened the Project by clicking on the project title, hit <Record Status Dashboard> on the LEFT panel to locate your 1.5T/3T phantom by ID number from the list of buttons.
6. If your center has received **more than one** phantom, please search for both IDs.
7. If your center plans to serially scan a particular phantom **on more than one magnet** for the 1-year period (e.g. you plan to scan your phantom '15E001' on both a 1.5T Philips **and** 1.5T GE) then please search for Phantom ID plus the Magnet Denomination (e.g. 15E001axr and 15D001sdx).
8. At first login, you will be asked to fill in more magnet-specific data. This first module is called <Enrolment> and it is launched by hitting the first 'Button' on the dashboard, nearest to your Phantom ID.
9. Some fields inside the <Enrolment> module **will be pre-filled** based on information you had previously submitted to us. Please check that all this information is still correct.
10. If your center has received more than one phantom or if you are scanning your phantom on more than one magnet, then please verify and complete the separate <Enrolment> pages that we have prepared for you.
11. If scanner hardware/software items change over the course of the year, or you undergo an upgrade or a service you will have a chance to refresh this information by entering it into the corresponding repeating forms for each of the serial scans.
12. Once you have completed a module select <COMPLETE> at the bottom of the form and then <SAVE>.
    a. <COMPLETE> turns button for that module GREEN.
    b. <INCOMPLETE> turns button for that module RED.
    c. <UNVERIFIED> turns button for that module YELLOW (e.g. If you need to check on a sequence detail/scanner date and enter that information later etc).
    d. An empty module, containing no data will appear GREY.
13. To the right of the <Enrolment> button, are many other GREY buttons called <SCAN_1> all the way to <SCAN_30>. As we hope that you will be scanning the T1MES phantom every fortnight for about a year, 30 repeating modules have been empirically created for your device.
14. Please note REDCap is NOT where you upload the scan DICOMs (see 6.2).
15. We shall however be uploading your phantom analysis results onto REDCap ourselves, inside each of your modules for your device sequentially.

16. After filling in the one-off <Enrolment> module at the start, you **WILL NOT** need to access REDCap for any data input after each repeat scan **UNLESS** it is to:
    a. report a software/hardware change/update
    b. report that you are having to make a change to the baseline T1 mapping sequence/s that you had previously been using (please avoid this as much as possible)
    c. report an issue with the phantom or any other aspect of our infrastructure
    d. download copies of your phantom's results or review pooled data
17. As you will already have entered <Temperature> and <Scan_Date> information per scan directly into the Phantom Registration ID, there is **NO NEED** to repeat this data entry procedure inside REDCap as well.
18. Therefore, provided none of the criteria in **16a-d** apply, **YOUR ONLY TASK** after phantom scanning will be to submit DICOMs through the SFTP portal, as outlined next in 6.2.

6.2 | DICOM PORTAL

1. You will have received an email and a separate instruction manual for how to download and install Filezilla SFTP to be able to access the **T1MES_DICOM_PORTAL**.
2. Once you click on the folder 'T1MES_DICOM_PORTAL' you will find a collection of >80 subfolders inside named according to Phantom ID and in some cases with an additional Magnet Denomination. Locate the subfolder/s for your phantom/s ID +/- magnet.
3. Each phantom subfolder then contains 30 further subfolders labeled SCAN_1 to SCAN_30 (matching the REDCap modules).
4. At the end of each fortnightly scan export all the DICOMS pertaining to the T1MES scan that has just been performed. Please make sure that you **do** export ***ALL*** the DICOMS (i.e. T1 maps but also any scouts, MOCO if on, T1 T1* residual etc).
5. **There is NO NEED to anonymise this data**.
6. Transfer the DICOMS into the correct Phantom ID and sequential SCAN subfolder (SCAN_1 > SCAN_2 > SCAN_3 etc.) so that we may start your analysis in a timely fashion.
7. Once we have processed the data pertinent to your latest submission (e.g. SCAN_1), we will be responsible for changing that subfolder's name in your DICOM_PORTAL to: "**x**SCAN_1".
8. Thus, you will easily know next time, to transfer DICOMS straight into SCAN_2 subfolder located at the top of your list.

7 | RESULTS

1. We will be providing prompt analyses of site data and displaying these graphically as well as providing stability data and potentially in future looking at conversion equations to generate standard T1 values.
2. We want to provide anonymised multisite data to show broad stabilities. These interfaces will be built once we have multisite data to look at.

8 | APPENDIX

A8.1 | SIEMENS-specific instructions

A8.1.1 | Table positioning of phantom for SIEMENS

1. For Siemens the bottle should be consistently positioned between the same table markers each time (e.g. always between **S1|S2 or S2|S3 marker levels** or if using the 32-channel posterior array consistently center on that array). This is not essential but we ask as this is easily replicated on each session in case of any unforeseen sources of variability (e.g. there is some metal in the patient table which is known to affect some sequences).
2. Our tests on a 3T Siemens Skyra with a detachable couch have suggested that a firm support of height 7.5cm provides the right height to achieve T1MES isocenter positioning along the *y* axis.
3. The height for your particular Siemens machine, table and couch settings ***may vary*** so please test firm support height to find your ideal setup (then remember it and if possible store it with phantom!).
4. If your firm support height is correct and isocentering by laser target markers was done carefully, you should expect to see transverse (axial) images similar to those in **Figure A1** when you open your T1 mapping sequence after running the scouts.
5. Aim for the middle of the T1MES phantom to be within +/-1cm of isocenter across the bed and within +/-3cm for height (but consistent to within +/-1cm each repeated session).

Figure A1. Confirmation of good isocentering of T1MES in Siemens (same for Philips).

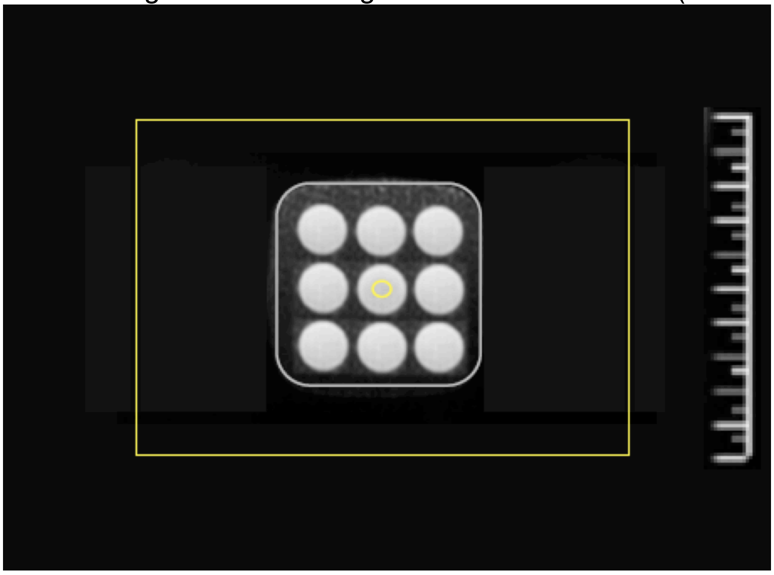

A8.1.2 | Register the phantom for SIEMENS

1. <Surname> enter your unique Phantom ID_Temp_Date of scan (with date as DDMMYYYY).
   e.g. **15E001_22_31012015** if temperature 22 degrees or
   e.g. **30E004_23_31012015** if temperature 23 degrees
   (**ONLY** for centers scanning **one phantom on more than one magnet**: remember to add the 3-letter <Magnet Denomination> after <Phantom ID> and before <Temperature>. Eg. for Siemens 3T Prisma Fit register as: **30E004pft_23_31012015**).

2. <Name> Copy paste same content as <Surname> (1) above.
3. <ID> Copy paste same content as <Surname> (1) above.
4. <DOB> 1/1/1980.
5. <Sex> Male.
6. <Height> 1.8 (m) or 180 (cm).
7. <Weight> 80 (kg).
8. <Patient position> select <Head first – supine>.
9. <Region being scanned> select <Heart> as this can affect specific absorption rate (SAR) modeling.
10. **Enable** <First-level SAR> and <First-level PNS "SP"> (predicted peripheral nerve stimulation) either during registration or if the scanner asks about these during phantom scanning. This is essential in case the T1 mapping sequence parameters are modified to lower these by "helpful" scanner software.

*A8.1.3 | Setting up simulated ECG for **SIEMENS***

1. You will need the Advanced User mode (password is typically <meduser1> for Siemens).
2. Start with <Ctrl-Esc> to launch the <Windows Start> menu.
3. Chose <Run> option.
4. Type: ideacmdtool
5. <Enter>
6. Type: 1
7. <Enter> to Start PMU (physiologic monitoring unit) control.
8. Type: 1 (or '4' on >VD-level software)
9. <Enter> to start ECG simulation.
10. For <ECG period in ms [1000]:>
11. Type: **900**
12. For <RESP period in ms [3000]:>
13. <Enter>
14. For <PULSE period in ms [1000]:>
15. Type: **900**
16. For <EXT period in ms [2000]:>
17. <Enter>
18. Check that the ECG waveform parameters generated are HR=**67**bpm and RR period =900ms.
19. You may now close the black "ideacmdtool" window (you do not need to remember to turn off the simulated ECG settings by using <ideacmdtool> again after the end of your phantom scans to allow the resumption of normal clinical scans, as a new <Patient Registration> will automatically clear it).

△ **FOR SIMULATED ECG PLEASE ALLOW TIME FOR SOFTWARE-"AVERAGED" HEART RATE** (if your system uses it) **TO SETTLE, BEFORE STARTING T1MAPPING**

*A8.1.4 | Shim settings for **SIEMENS***

1. Navigate to <System>
2. <Adjustments>
3. Next to <B0 Shim mode> choose the option <Cardiac>
4. If you do not have the option <Cardiac> available on your Siemens system, then choose <Standard>
5. Make sure that the option <Adjust with body coil> is **ticked**.

*A8.1.5 | Adjust volume for **SIEMENS***

1. Go to <System> and then to <Adjust Volume>.
2. Next to <! Position> select from dropdown list <Isocenter>.
3. Please apply the shim volume tightly around the phantom bottle as shown in **Figure A2**. It should be set 5cm thick in the HF direction and it should follow (+/-2cm) the size of the T1MES bottle in the RL and antero-posterior (AP) directions. Adjust the size of the green volume as follows:
    a. Set AP to **120** (mm)
    b. Set RL to **120** (mm)
    c. Set HF **thickness** of the shim slab to **50** (mm).
4. Prescribe the size of the volume in the 3 orthogonal planes to ensure consistent adjustments of B_0 and scanner reference frequency over the phantom each time you scan it.
5. Acquire the T1 mapping sequence at isocenter with the T1MES phantom right in the middle of the adjust volume.

Figure A2. Applying the adjust volume over T1MES for Siemens (same for Philips).

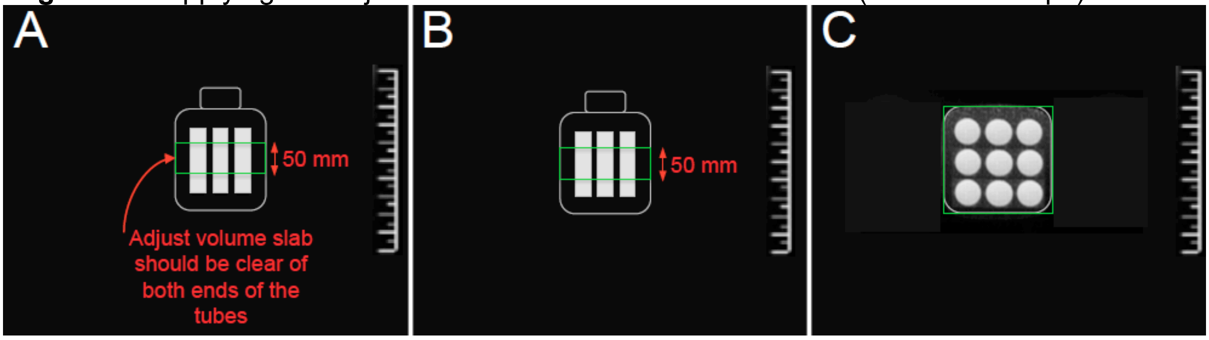

A8.2 | **PHILIPS**-specific instructions

*A8.2.1 | Table positioning of phantom for **PHILIPS***

1. The T1MES isocenter cross should be placed at the level of the MIDDLE side marker on the Philips table (there are side markers denoting the top, middle and bottom of the elements encased in the coil base).
2. Our tests on a 3T Philips Achieva have suggested that a firm support of height **8cm** provides the right height to achieve T1MES isocenter positioning along the *y* axis.
3. The height for your particular Philips machine, table and couch configuration ***may vary*** so please test firm support height to find your ideal setup (then remember it and if possible store it with phantom!).
4. If your firm support height is correct and isocentering by laser target markers was done carefully, you should expect to see images similar to those in **Figure A1** (in previous **section A8.1.1**) when you open your T1 mapping sequence after running the scouts.

*A8.2.2 | Register the phantom for **PHILIPS***

1. Select <Patient> on the main menu bar.
2. Select <New exam>.
3. Patient name: enter your unique Phantom ID_Temp_Date of scan (with date as DDMMYYYY).
   e.g. **15E001_22_31012015** if temperature 22 degrees or
   e.g. **30E004_23_31012015** if temperature 23 degrees.

(**ONLY** for centers scanning **one phantom on more than one magnet**: remember to add the 3-letter <Magnet Denomination> after <Phantom ID> and before <Temperature>. Eg. for Philips 1.5T Achieva XR register as: **15D001axr_22_31012015**).

4. Registration number (also called Registration ID): Copy + Paste same content as <Patient Name> (1) above.
5. Patient birth date: 01/01/1980.
6. Patient's sex: Male.
7. Weight: 80 (kg).
8. Referring physician: Phantom.
9. Comments: leave blank.
10. <Enter>.
11. Click <Proceed> at the bottom of the panel.

*A8.2.3 | Setting up simulated ECG for **PHILIPS***

1. If using the MOLLI patch[2] on the latest MR software (release 5) refer to footnote for quick ECG setup instruction.
2. Right click the main window and select <Control Parameter Editor>.
3. Select <General Tab>.
4. Select <Physiology Simulation>.
5. Select <Yes>.
6. Set the number of RR intervals = 1
7. Set RR interval (in ms) = **900**
8. Click <Apply>.
9. Remember to turn off the simulated ECG settings after the end of your phantom scans to allow the resumption of normal clinical scans.

△ **FOR SIMULATED ECG PLEASE ALLOW TIME FOR SOFTWARE-"AVERAGED" HEART RATE** (if your system uses it) **TO SETTLE, BEFORE STARTING T1MAPPING**

*A8.2.4 | Before running a T1 Map that involves SENSE in **PHILIPS***

1. Before setting up your mapping protocol it is your responsibility to check the control parameters of your Philips platform.
2. Ensure control parameters on Philips are set to DEFAULT VALUES.
3. Ensure no "patches" that could impact scanner behaviour unexpectedly are installed, except of course the patch to run your T1 mapping sequence if you need this.
4. Allow the scanner to perform the SENSE/CLEAR reference scans if using a T1 mapping sequence with parallel imaging (as the vast majority do).
5. If it asks about **PPNS** and **SAR** allow 1st Level.

*A8.2.5 | Shim settings for **PHILIPS***

1. To set shim mode on Philips select <Contrast> tab.
2. Locate <Shim> in the column of parameters on the left.
3. Select option <volume>. **CAUTION** - please do not use other "shim" options and if in doubt please ask for advice.
4. Next adjust the green volume slab: Please apply the shim volume tightly around the phantom bottle as shown in **Figure A2** (in previous **section A8.1.5**). It should be set 50mm thick in the HF direction and it should follow (+/-2cm) the size of the T1MES bottle in the RL and AP directions.

---

[2] *Go to main interface > Motion Tab > Next to 'Cardiac simulation' select 'yes' then set your RR interval to 900ms.*

5. Go to the tab <offc/ang>.
6. Under <Shim Size> adjust the size of the green volume as follows:
    a. Set AP to **120** (mm)
    b. Set RL to **120** (mm)
    c. Set HF **thickness** of the shim slab to **50** (mm).
7. Prescribe the size of the volume in the 3 orthogonal planes to ensure consistent shim and reference frequency estimation over the phantom each time you scan it.
8. Acquire the T1 mapping sequence at isocenter with the T1MES phantom right in the middle of the adjust volume.

A8.3 | GE-specific instructions

A8.3.1 | Table positioning of phantom for GE

1. Our tests on a 3T GE 750 have suggested that a firm support of height **3cm** provides the right height to achieve T1MES isocenter positioning along the *y* axis.
2. The height for your particular GE machine, table and couch configuration ***may vary*** so please test firm support height to find your ideal setup (then remember it and if possible store it with phantom!).
3. If your firm support height is correct and isocentering by laser target markers was done carefully, you should expect to see images similar to those in **Figure A3** when you open your T1 mapping sequence after running the scouts.
4. Aim for the middle of the T1MES phantom to be within +/-1cm of isocenter. If it's perfectly correct you will see an <X> mark in the middle tube on the GE scanning software. A +/-3cm offset on the vertical (*y*) axis is acceptable but whatever height is achieved should be replicated each session to within +/-1cm. Both horizontal directions (across [*x*] and along [*z*] the patient bed) should be easily within +/-1cm using the laser guides and isocenter cross sticker.

Figure A3. Confirmation of good isocentering of T1MES in GE.

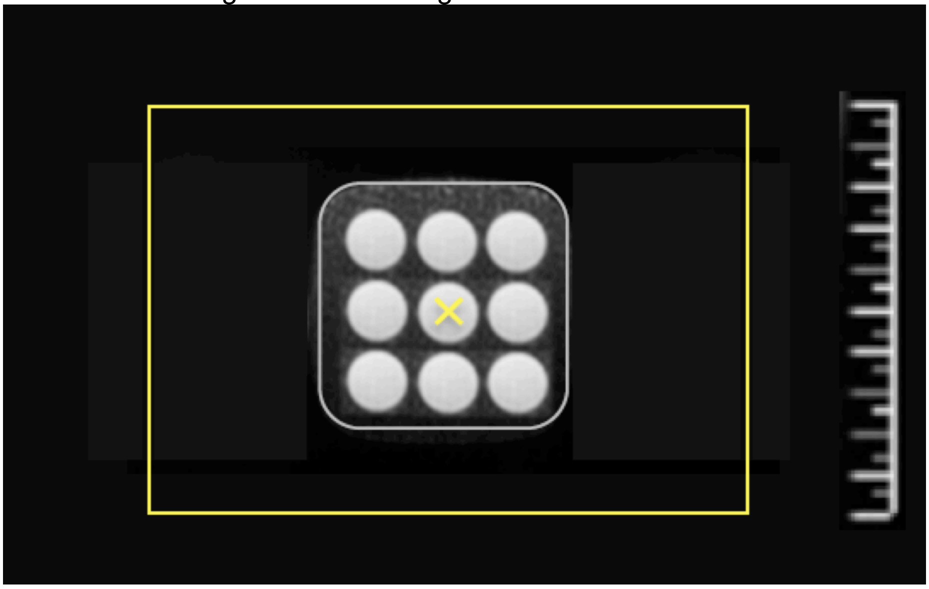

A8.3.2 | Register the phantom for GE

1. Select <Scan> desktop icon.
2. Click <New Pt> from the Patient Register window.

3. Patient ID: enter your unique Phantom ID_Temp_Date of scan (with date as DDMMYYYY).
   e.g. **15E001_22_31012015** if temperature 22 degrees or
   e.g. **30E004_23_31012015** if temperature 23 degrees.
   (**ONLY** for centers scanning **one phantom on more than one magnet**: remember to add the 3-letter <Magnet Denomination> after <Phantom ID> and before <Temperature>. Eg. for GE 1.5T Signa HDx register as: **15D001sdx_23_03022015**).
4. Patient name: Copy + Paste same content as ‘Patient ID’ (3).
5. Birth date: 01/01/1980.
6. Sex: Male.
7. Weight: 80 (kg).

*A8.3.3 | Setting up simulated ECG for **GE***

1. **OUR PREFERENCE** is that you use (or purchase, if you do not already have one) **a basic ‘ECG simulator’ (also known as ‘Chicken Heart’)**. Work with CAUTION this ECG simulator may be dangerous in the magnet room.
2. Set heart rate on the device to **67bpm**.
3. Please position the ECG simulator a safe distance from the bore and follow the manufacturer’s instructions.
4. The **less preferred** alternative is to use the command window to set the ECG Simulator on GE to 80bpm.
5. Go to <Tools> menu (this is the icon at the top left showing a spanner/hammer).
6. Click on the icon’s arrow to reveal the drop down.
7. Select <Command Window>.
8. Type: rlogin scp
9. <Enter>
10. Login: scp
11. Password: scpservice
12. Type: SCP_LX->EmulatePac
13. <Enter>
14. This sets a simulated ECG with rate 100bpm (this heart rate is too fast for the T1 mapping planned for T1MES. Siemens and Philips users will be using a simulated ECG of 67bpm).
15. To slow down the heart rate (imperfectly) go to back to <Tools> menu and hit the actual button.
16. Select <Open Gating Control Window>.
17. <Waveform display> (select all).
18. By setting <Trigger Lead> to <PG> heart rate will slow from 100bpm to 80bpm – it is still imperfect as the heart rate of 67bpm cannot be achieved; hence why we recommend the ‘Chicken Heart’. Either way, please use the same heart rate for all sessions!
19. If you have set the simulated ECG using the command window in GE, please allow time for any software-“averaged” heart rate (if your system uses it) to settle, before starting T1 mapping work.
20. If you have set the simulated ECG using the command window in GE, remember to disable it when done scanning to permit resumption of routine clinical scans. To disable simulated ECG: hit <Tools> icon.
21. Select <Go to Service Desktop Manager> and hit <TPS Reset> to REMOVE the simulated ECG and reset the gating.

A8.3.4 | Before running a T1 Map that involves SENSE in ***GE***

1. Select to run <**First Level Mode**> if prompted.

A8.3.5 | Shim for ***GE***

1. Shim volume in GE is shaped by default as an obligatory cube.
2. To adjust its size go to <Graphic Rx Toolbar> (if you do not immediately see the Toolbar, hit the <GRx> tab).
3. Click <Shim>.
4. Uncheck the <Hide Shim> option to reveal the shim cube (it will look like a green/yellow hashed cube).
5. Next to <FOV> enter <10> to set the size of the cube to 10 X 10 X 10cm.
6. Move the cube so that it covers the middle lengths of the tubes and try to avoid the edges at either end as in **Figure A4**.

Figure A4. Applying the adjust volume over T1MES for GE.

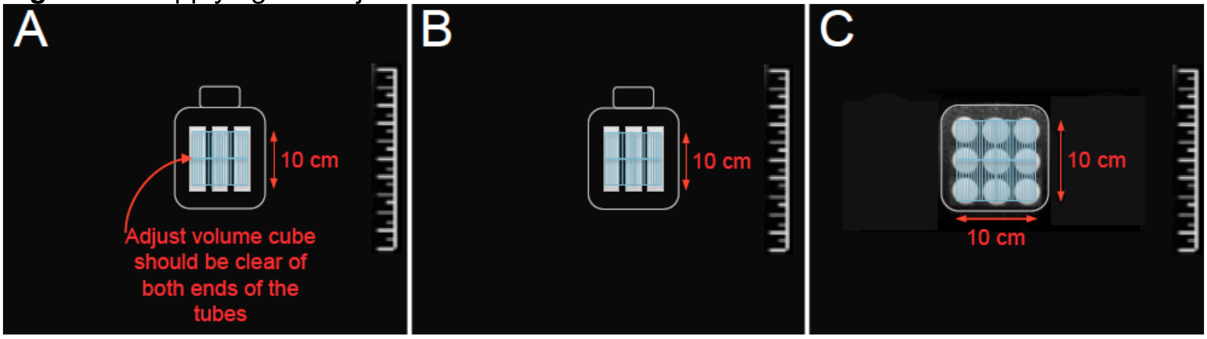

Thank you
